# Rice‐derived SARS‐CoV‐2 glycoprotein S1 subunit vaccine elicits humoral and cellular immune responses

**DOI:** 10.1111/pbi.70077

**Published:** 2025-04-04

**Authors:** Li Song, Yaya Wen, Yu Zhou, Hui Zhang, Yuqi Tian, Jing Wang, Yaodan Cui, Ruimeng Tan, Dan Xiong, Chuang Meng, Yan Zhou, Qianfeng Li, Zhiming Pan, Qiaoquan Liu, Xinan Jiao

**Affiliations:** ^1^ Jiangsu Key Laboratory of Zoonosis, Jiangsu Co‐innovation Center for Prevention and Control of Important Animal Infectious Diseases and Zoonoses, Key Laboratory of Prevention and Control of Biological Hazard Factors (Animal Origin) for Agrifood Safety and Quality of the Ministry of Agriculture and Rural Affairs of China, Joint International Research Laboratory of Agriculture and Agri‐product Safety of the Ministry of Education, Yangzhou University Yangzhou China; ^2^ Jiangsu Key Laboratory of Crop Genomics and Molecular Breeding, Key Laboratory of Plant Functional Genomics of the Ministry of Education, College of Agriculture and Co‐Innovation Center for Modern Production Technology of Grain Crops of Jiangsu Province Yangzhou University Yangzhou China

**Keywords:** transgenic rice, SARS‐CoV‐2, rice‐derived S1 protein, subunit vaccine, immunogenicity, humoral and cellular immune responses

## Abstract

Since 2019, severe acute respiratory syndrome coronavirus 2 (SARS‐CoV‐2), the virus causing COVID‐19, has been spreading and mutating globally despite the expedited approval of many commercial vaccines. Therefore, developing safe, effective and affordable vaccines remains essential to meet the global demand, particularly in developing countries. Transgenic plants have emerged as a promising platform to express recombinant proteins for pharmaceutical and vaccine applications. Two binary vectors, pCAMBIA1300Gt1‐S1 and pCAMBIA1300Actin‐S1, containing distinct promoters, were constructed and transformed into rice via *Agrobacterium*. Overall, 56 independent transgenic rice lines were regenerated. Expression analysis revealed that the rice‐derived S1 (rS1) protein could be expressed in pGt1::S1 transgenic rice seeds. rS1 protein expression levels reached up to 282 μg/g dry weight, with S1 gene insertion having no effect on grain size and weight. The rS1 protein exhibited a high affinity for human angiotensin‐converting enzyme 2 (ACE2) *in vitro*. Moreover, the immunogenicity of purified rS1 protein co‐administered with various adjuvants demonstrated that mice vaccinated with Alum‐adjuvant rS1 generated enhanced humoral immune responses with high serum IgG, IgG1 and neutralizing antibody levels. *Salmonella* Typhimurium flagellin (FliC)‐adjuvanted rS1 elicited stronger S1‐specific IgG2a levels, promoted splenocyte proliferation and induced mixed Th1/Th2/Th17 cytokine responses. This was evidenced by increased proportions of antigen‐specific interferon (IFN)‐γ, interleukin‐4 (IL‐4) and IL‐17A‐positive CD4^+^ T lymphocytes, suggesting its potential to induce both humoral and cellular immune responses. These findings suggest that rS1 protein offers a promising approach for affordable COVID‐19 subunit vaccine production, and this strategy can be universally applied to other viral vaccines.

## Introduction

Coronavirus disease 2019 (COVID‐19), a highly contagious global pandemic caused by severe acute respiratory syndrome coronavirus 2 (SARS‐CoV‐2), remains endemic worldwide, with frequent variant‐driven outbreaks despite the urgent approval of multiple commercial vaccinations. As of 4 August 2024, over 775 million incidences and 7.0 million COVID‐19‐related mortalities have been reported globally (World Health Organization, 2024). Developing vaccines that induce effective immune responses while ensuring safety and affordability remains a critical challenge.

SARS‐CoV‐2, an enveloped, positive‐strand RNA virus, belongs to the *Betacoronavirus* genus (Pal *et al*., 2020). The coronavirus genome encodes several viral proteins, including spike (S), envelope (E) and membrane (M) proteins (Lu *et al*., 2020). The S protein is essential for viral entry into host cells and can elicit effective immune responses after vaccination (Walsh *et al*., 2020). Each S protein monomer comprises an S1 subunit as the globular head and an S2 subunit located near the membranes. SARS‐CoV‐2 binds to host cells through the receptor‐binding domain (RBD) on S1, which interacts with human angiotensin‐converting enzyme 2 (ACE2) in susceptible cells (Huang *et al*., 2020). As the primary target for virus‐neutralizing antibodies (NAbs), the S1 alone is protective in animal models and is a central focus of vaccine design (Watanabe *et al*., 2020).

Various vaccine strategies have been explored, including plasmid DNA, adenovirus vectors, protein subunits and inactivated viruses, among which subunit vaccines possess superior safety (Strauch *et al*., 2017). Plants provide an appealing platform for antigen production (Margolin *et al*., 2018) owing to their easy scalability, low production costs, high‐quality yields, absence of contamination and process of eukaryotic protein modification (He *et al*., 2011). Furthermore, Plant‐based vaccines avoid virus and bacteria cultures, eliminating infection risk and making molecular farming particularly advantageous for developing countries (Fausther‐Bovendo and Kobinger, 2021). Several plant‐based vaccines targeting viruses, such as hepatitis C, porcine circovirus type 2 and influenza A H6N2, have been explored, highlighting the significant potential of plant‐based biopharmaceuticals (Dobrica *et al*., 2021; Gunter *et al*., 2019; Smith *et al*., 2020). In the context of COVID‐19 plant‐based vaccines, the coronavirus virus‐like particle (CoVLP) vaccine expressed in *Nicotiana benthamiana* has received emergency use authorization (Charland *et al*., 2022; Jung *et al*., 2022). Meanwhile, vaccines produced in plants such as rice (Saba‐Mayoral *et al*., 2023; Sobrino‐Mengual *et al*., 2024) and lettuce chloroplasts (Singh *et al*., 2023) remain under development.

The ‘ideal vaccine’ should be effective, safe, cost‐effective, readily scalable for mass production and easily stored and transported. Seed‐based expression systems present promising options for producing recombinant proteins (Boothe *et al*., 2010). Rice seeds serve as a cost‐effective bioreactor for large‐scale production (He *et al*., 2011). However, recombinant protein vaccines require adjuvants to trigger effective and robust immune responses (Jangra *et al*., 2021). Therefore, developing adjuvants is crucial for advancing subunit vaccines against COVID‐19. Toll‐like receptor (TLR) signalling could drive T cell responses and maturation of antibodies against viruses. TLR5 is expressed on innate immune cell surfaces in various species (Iwasaki and Medzhitov, 2010). Our previous research shows that the combination of HA1‐2 antigen from H7N9 and TLR5‐agonist FliC induces robust immune responses against the influenza virus (Song *et al*., 2017). Moreover, vaccines using flagellin as adjuvants provide protection in mice and non‐human primate models (Cui *et al*., 2018).

In this study, two binary vectors containing the S1 gene of SARS‐CoV‐2, controlled by a tissue‐specific promoter (rice glutelin, Gt1) and a constitutive promoter (Actin), were constructed. These vectors were introduced into rice by *Agrobacterium*‐mediated transformation. The expression and ACE2 binding activity of rice‐derived S1 (rS1) protein were assessed *in vitro*, and its immunogenicity was evaluated in a mouse model. This study aims to investigate a plant‐based vaccine utilizing rice‐derived S1 protein to elicit robust immune responses against SARS‐CoV‐2.

## Results

### Screening of homozygous S1 transgenic plants

Two recombinant plasmids (pGt1‐S1 and pActin‐S1) were constructed and separately introduced into the rice variety ZH11 using *Agrobacterium*‐mediated transformation. Overall, 56 independent T_0_‐positive transgenic rice plants were obtained. Subsequently, stable expression lines of transgenic rice were screened using PCR analysis. Following segregation analysis of the T_1_ generation progeny (T_1_‐1 to T_1_‐56), several pGt1::S1 transgenic plants exhibiting a segregation ratio of approximately 3:1 were selected for further self‐crossing to generate T_2_ transgenic plants. To ensure an adequate seed supply for subsequent applications, six T_2_ pGt1::S1 transgenic plants exhibiting 100% positivity for the target gene were selected for the production of T_3_ transgenic plants (Figure S2b and Table S3). To further elucidate the homozygosity of the T_3_ generation, segregation analysis was conducted by evaluating hygromycin resistance. Unlike the non‐transgenic control and heterozygous plants, all T_4_ plants survived the hygromycin selection, demonstrating 100% resistance (Figure S3 and Table S4), thereby confirming that the T_3_ plants (T_3_‐1 to T_3_‐6) were homozygous.

### Identification of rS1 protein in rice seeds and leaves

**Figure 1:**
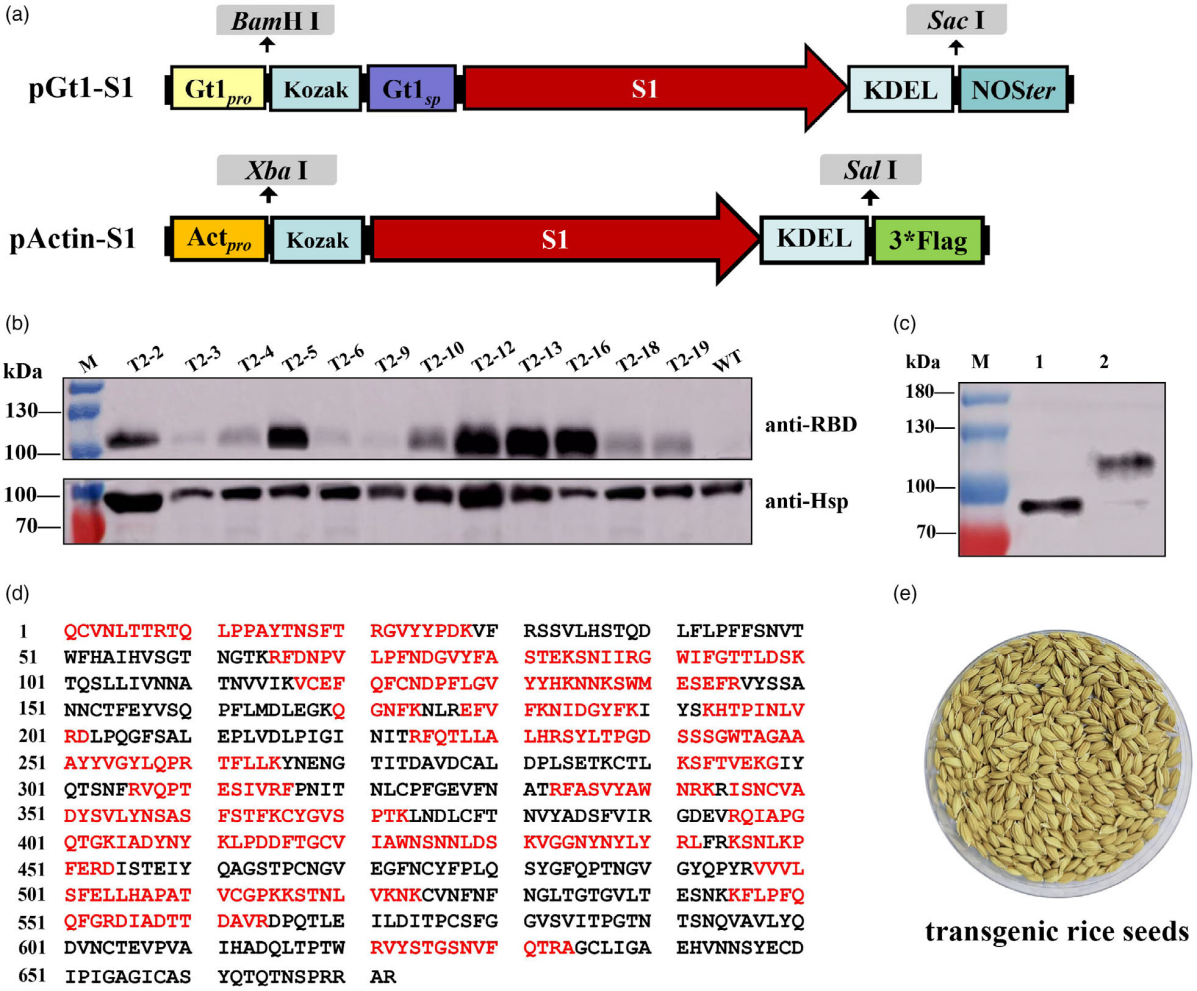
Genetic construction and detection of rS1 protein. (a) Schematic diagram of the recombinant S1 antigen design. Gt1_pro_: Gt1 Promoter, a rice seed storage protein glutelin gene promoter; Kozak sequence: Sequence enhancing translation initiation; Gt1_sp_: Gt1 Signal peptide; Codon‐optimized S1 gene: Sequence adapted for expression in rice; KDEL: Endoplasmic reticulum retention signal; NOS_ter_: Nopaline synthase gene terminator; Act_pro_: Actin promoter, a constitutive promoter. (b) Expression of the rS1 protein in T_2_ transgenic rice seeds. Total protein from T_2_ generation transgenic rice seeds was extracted for rS1 protein identification using Western blot analysis. Hsp protein was used as the internal control for gene expression normalization. M: Protein marker; WT: Non‐transgenic control. (c) N‐glycosylation modification of the rS1 protein. M: Protein marker; 1: T_2_‐2 Transgenic rice seed extract; 2: T_2_‐2 Transgenic rice seed extract treated with PNGase F. (d) Mass spectrometry analysis of the rS1 protein. Matched peptides in the S1 protein are highlighted in Bold Red. (e) Representative diagram of homozygous seeds from T_2_ transgenic rice.

The expression of rS1 in transgenic seeds and leaves was analysed using Western blot analysis. For the pGt1::S1 transgenic lines, a specific band of approximately 110 kDa was observed in the S1 transgenic rice seeds, while no such band was detected in the ZH11 WT seeds. These bands were specifically recognized by antibodies against the RBD protein (Figure 1b and Figure S4d). However, no specific bands were observed in the seeds and leaves of the pActin::S1 transgenic lines compared to the WT rice plants (Figure S5). Furthermore, the results showed that the molecular weight of the rS1 protein extracted from pGt1::S1 transgenic seeds was approximately 76 kDa after PNGase F treatment (Figure 1c). This finding was consistent with expectations, indicating that N‐glycosylation modifications occurred during the expression of the S1 protein in the rice endosperm. To further confirm the expression of the rS1 protein, mass spectrometry (MS) analysis was performed. The results showed that 51% of the S1 amino acid sequence was detected (Figure 1d). These results demonstrated that the rS1 protein was successfully expressed in the pGt1::S1 transgenic rice seeds, while no expression was detected in the pActin::S1 transgenic rice.

### Expression levels of rS1 protein in rice seeds

**Figure 2:**
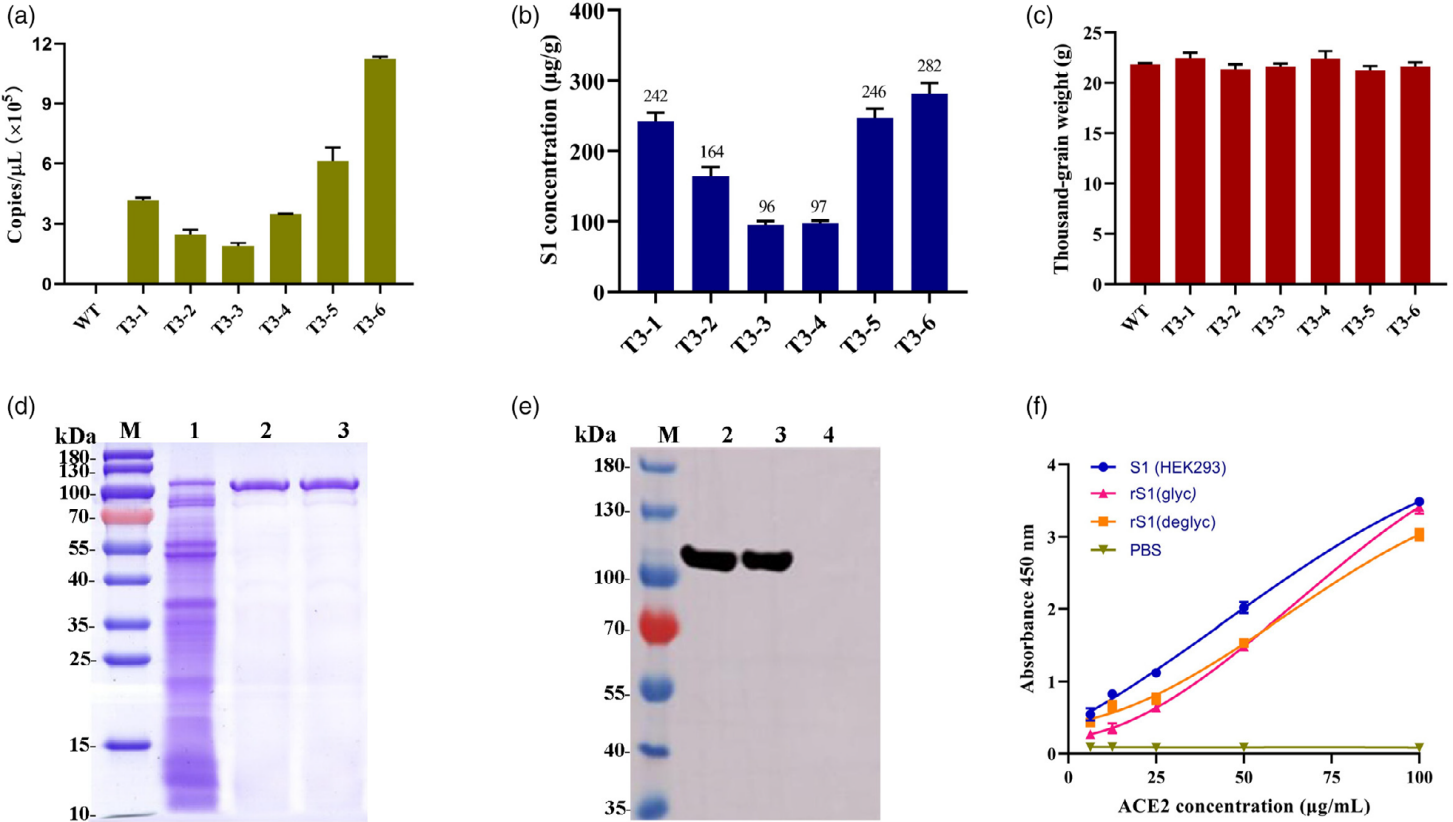
Quantification and purification of rS1 protein. Developing seeds (T_3_ generation) were collected 15 days after flowering (DAF) to analyse the transcription levels of the S1 gene. Total protein was extracted from mature T_3_ generation rice seeds and the rS1 protein concentration in the extract was measured using a quantitative Western blot assay. (a) mRNA levels of the S1 gene in different transgenic lines and the non‐transgenic control (WT). (b) rS1 concentration in T_3_ generation transgenic rice seeds. (c) Thousand‐grain weight (TGW) of T_3_ generation transgenic rice seeds and the WT. (d) SDS‐PAGE of purified rS1 protein. (e) Western blot results of purified rS1 protein. M: Protein marker; 1: Extract from T_3_ transgenic seeds; 2–3: Purified rS1 protein; 4: Extract from non‐transgenic control (WT). (f) ACE2 binding activity of the S1 proteins. rS1 (glyc): purified rS1 protein; rS1 (deglyc): rS1 protein treatment with PNGase F; S1 (HEK293): S1 protein expressed in HEK293 cells.

The transcriptional levels of the rS1 protein in homozygous rice lines (T_3_ generation) were measured using probe‐based qRT‐PCR. The copy numbers of the S1 gene were calculated using a standard curve generated from an S1‐containing plasmid of known concentrations (Figure S4a,b). Among the six homozygous lines, the T_3_‐6 transgenic plant exhibited the highest number of S1 gene copies in its seeds, approximately 1.12 × 10^6^ copies/μL (Figure 2a).

The expression levels of the rS1 protein in homozygous rice lines were measured using quantitative Western blot analysis. The concentration of the rS1 protein was calculated based on a standard curve. A linear dose–response curve was established over the range of 1.25–20 μg/mL for the standard S1 protein, with a coefficient of determination (R^2^) of 0.9952 (Figure S6c). The results showed that the rS1 antigen accumulated to levels ranging from 96 to 282 μg/g of dry weight. Of the six lines tested, the transgenic rice line T_3_‐6 exhibited the highest expression level at 282 μg/g (Figure 2b and Figure S6), which is consistent with the findings from the qRT‐PCR analysis. The percentage of rS1 protein within the total seed protein varied from 2.7% to 6.3% (Figure S6d). Based on these results, the T_3_‐6 transgenic rice line was selected for further study.

### Rice grain size and thousand‐grain weight analysis

To evaluate the effect of S1 expression on rice seeds, the grains (T_3_ generation lines 1–6) were collected for the investigation of agronomic traits. No significant differences in grain length, grain width or thousand‐grain weight (TGW) were observed in the seeds of the transgenic plants compared to the WT control (Figure 2c and Figure S7). This finding suggests that the S1 gene insertion does not affect the size and weight of rice seeds.

### Purification and ACE2 binding activity of rS1 protein

The T_3_‐6 transgenic rice seeds, exhibiting the highest expression levels, were used for protein purification. The results showed that the purified rS1 protein had a molecular weight of approximately 110 kDa, as observed in SDS‐PAGE (Figure 2d). The purified protein demonstrated strong and specific reactivity with rabbit anti‐RBD protein polyclonal antibodies (Figure 2e), indicating good immunoreactivity. Furthermore, the binding activity of the S1 protein to the ACE2 protein was analysed using an ELISA. The results showed that the binding affinity of S1 proteins to ACE2 increases with the concentration of ACE2. The rice‐derived S1 (rS1 glyc) exhibited weaker binding compared to HEK293‐produced S1, but its binding affinity approached that of HEK293‐produced S1 at elevated ACE2 concentrations (100 μg/mL). Furthermore, deglycosylated rS1 demonstrated stronger binding at lower ACE2 concentrations (<50 μg/mL), whereas glycosylated rS1 was more effective at higher concentrations (>50 μg/mL) (Figure 2f). These findings confirmed the successful purification of the rice‐derived S1 protein and provided preliminary validation of its biological activity.

### Humoral immune responses triggered by rS1 vaccine

**Figure 3:**
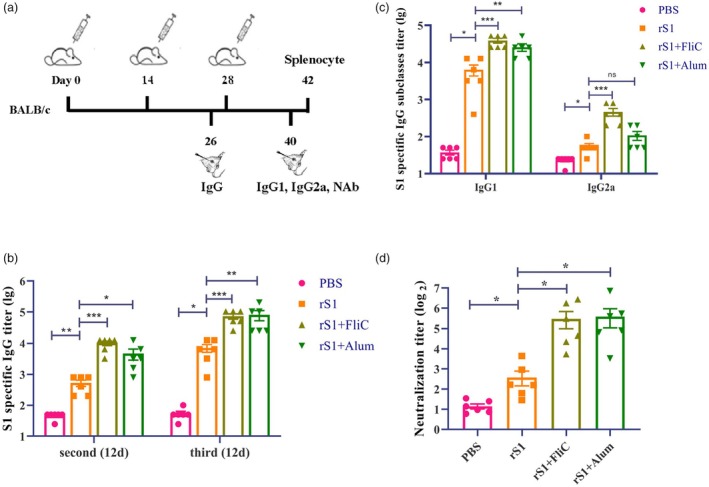
Humoral immune responses induced by rS1 protein. (a) Schematic illustration of the immunization and sampling procedure. Mice were intramuscularly immunized with three doses of rS1, rS1 + FliC, rS1 + Alum or PBS. Serum samples were collected on day 12 after the second and third immunisations to analyse antibody immune responses. (b) S1‐specific IgG titres in the serum on day 12 after the second and third immunisations. (c) S1‐specific IgG subtypes (IgG1, IgG2a) titres in serum on day 12 after the third immunization. (d) Neutralizing antibody titres in serum on day 12 after the third immunization. S1‐specific IgG, IgG1 and IgG2a titres were analysed using log10‐transformed data. Neutralizing antibody titres were analysed using log2‐transformed data. The data were presented as mean ± SEM from six mice per group. *P < 0.05, **P < 0.01 and ***P < 0.001 were considered statistically significant.

To assess the immunogenicity of rS1, BALB/c mice were immunized intramuscularly with three doses of rS1 protein, adjuvanted with FliC, Alum or administered unadjuvanted, at 2‐week intervals. After 2 weeks of the second or third vaccination, significantly higher titres of S1‐specific IgG antibodies were observed in response to rS1 than to the PBS. Furthermore, the FliC‐ and Alum‐adjuvanted rS1 induced significantly higher levels of IgG antibody than the rS1 administered alone. Additionally, rS1 induced titres of S1‐specific IgG that were 10‐fold higher following the third vaccination than the second vaccination (Figure 3b). Furthermore, the Alum‐adjuvanted rS1 induced a significantly higher IgG1 antibody response, while the FliC‐adjuvanted rS1 vaccine induced significantly increased IgG1 and IgG2a antibody responses compared to rS1 administered alone (Figure 3c).

### rS1 subunit vaccine triggers strong neutralizing antibody responses

To further investigate the efficacy of the antibodies generated by the rS1 vaccine, the serum samples from immunized mice were collected for a neutralization assay using SARS‐CoV‐2 pseudovirus. The results revealed that significantly higher NAb titres were observed in the sera of the rS1 + FliC and rS1 + Alum groups than in the rS1 alone group (Figure 3d). These data showed that the sera from mice immunized with the adjuvanted rS1 vaccines effectively neutralized the SARS‐CoV‐2 pseudovirus.

### Cellular immune responses triggered by rS1 vaccine

**Figure 4:**
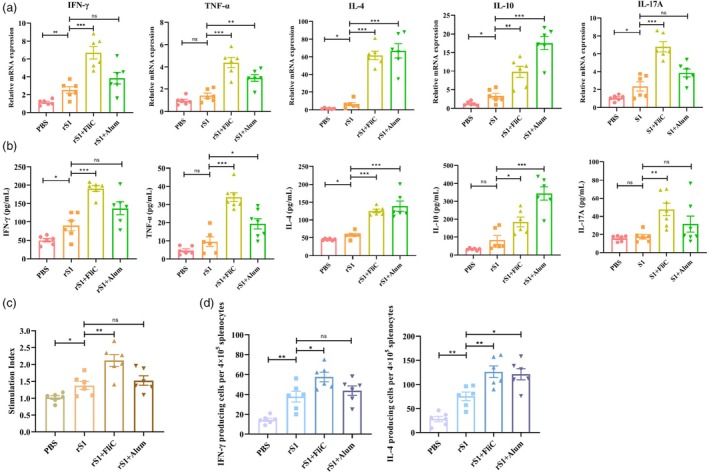
Cellular immune responses induced by rS1 protein. Mice were intramuscularly immunized with three doses of rS1, rS1 + FliC, rS1 + Alum or PBS. Spleens were harvested 2 weeks after the last vaccination and splenocytes were isolated and treated using 5 μg/mL of rS1 protein to assess cellular immune responses. (a) Expression levels of Th1, Th2 and Th17 cytokines (IFN‐γ, TNF‐α, IL‐4, IL‐10 and IL‐17A) measured using qRT‐PCR. (b) Secretion levels of Th1, Th2 and Th17 cytokines (IFN‐γ, TNF‐α, IL‐4, IL‐10 and IL‐17A) in cell supernatants measured using ELISA. (c) Splenocyte proliferation measured using a BrdU‐based ELISA kit. (d) IFN‐γ and IL‐4 secretion by splenic lymphocytes stimulated with S1 determined using ELISpot. The data were presented as mean ± SEM from six mice per group. *P < 0.05, **P < 0.01 and ***P < 0.001 were considered statistically significant.

The Spleens were harvested from mice 2 weeks after the third vaccination, while the cellular immune responses induced by the vaccination were evaluated through cell proliferation and cytokine expression assays. The mRNA levels of Th1 cytokines (IFN‐γ and TNF‐α), Th2 cytokines (IL‐4 and IL‐10) and Th17 cytokine (IL‐17A) in response to the S1 antigen were measured. The results demonstrated a significant increase in the expression levels of IL‐4 and IFN‐γ in the rS1 group compared to the PBS group. When rS1 was co‐delivered with Alum, the expression levels of IL‐4 and IL‐10 were significantly increased, while the expression of IFN‐γ and IL‐17A showed no significant difference. In contrast, when rS1 was co‐delivered with FliC, the expressions of IFN‐γ, TNF‐α, IL‐4, IL‐10 and IL‐17A were significantly higher than those in the rS1 group (Figure 4a). In addition, we quantified cytokine levels secreted by splenocytes following a two‐day restimulation with the rS1 protein. The rS1 group demonstrated significantly elevated levels of IL‐4 and IFN‐γ compared to the PBS control group. Furthermore, the rS1 combined with FliC group exhibited an increased secretion of all measured cytokines relative to the rS1 group. The group adjuvanted with Alum showed heightened levels of Th2 cytokines (IL‐4 and IL‐10); however, IL‐17A secretion was not elevated in either the rS1 or rS1 + Alum groups (Figure 4b). These findings were consistent with the results obtained from qRT‐PCR analysis. Additionally, the level of splenic lymphocyte proliferation in the rS1 + FliC group was significantly higher than that in the rS1 + Alum group (Figure 4c).

### Number of spleen lymphocytes secreting IFN‐γ and IL‐4 cytokines

The number of spleen lymphocytes secreting IFN‐γ and IL‐4 cytokines was detected using the ELISpot assay after the third immunization. The results showed that the numbers of spleen lymphocytes secreting IFN‐γ and IL‐4 in the rS1 group were significantly higher than those in the PBS group. Additionally, the levels in the S1 + FliC immune group (72; 143) were significantly higher than those in the S1 immune group (48; 89), indicating that the co‐delivery of rS1 with FliC enhances the ability of the rS1 protein to induce a balanced Th1/Th2 immune response. The number of spleen lymphocytes secreting IL‐4 in the S1 + Alum immune group (120) was significantly higher than that in the rS1 immune group (89), while the number of lymphocytes secreting IFN‐γ did not significantly differ from that in the rS1 immune group (Figure 4d).

### Cytokine‐producing CD4 ^+^ and CD8 ^+^ T cell responses

**Figure 5:**
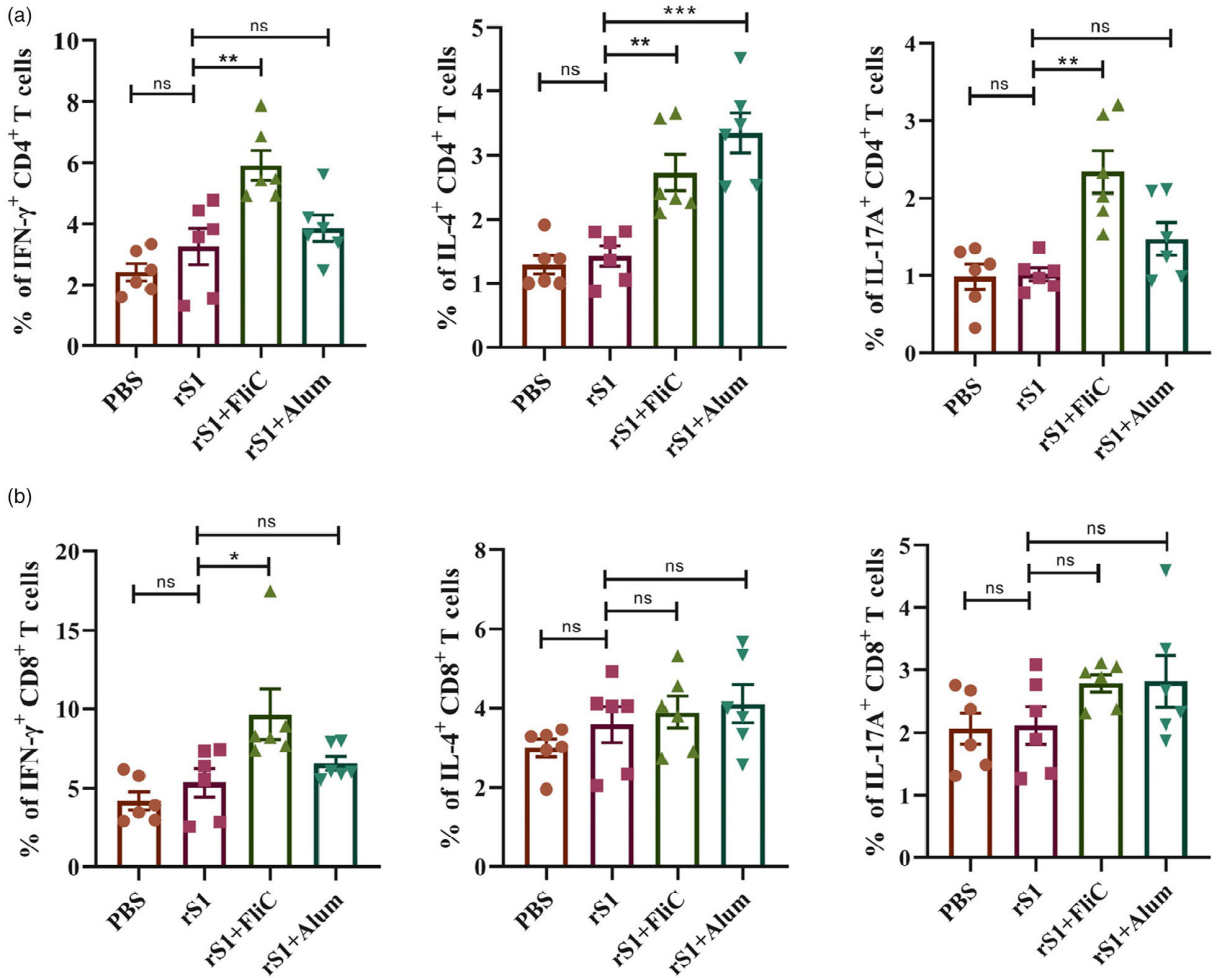
Cytokine‐producing CD4^+^ and CD8^+^ T cell responses. Splenocytes from vaccinated mice were harvested 2 weeks after last immunization and restimulated with rS1 protein for 6 h. Cytokine‐producing CD4^+^ and CD8^+^ T cell responses were detected by intracellular staining and quantified by flow cytometry. (a) IFN‐γ^+^, IL‐4^+^ and IL‐17A^+^ CD4^+^ T cells quantified within the CD4^+^ T cell population. (b) IFN‐γ^+^, IL‐4^+^ and IL‐17A^+^ CD8^+^ T cells quantified within the CD8^+^ T cell population. The results were expressed as the percentage of positive cells. The data were presented as mean ± SEM from six mice per group. *P < 0.05, **P < 0.01 and ***P < 0.001 were considered statistically significant.

Two weeks post the third vaccination, splenocytes from all vaccinated mice were restimulated ex vivo with the rS1 protein and cytokine‐producing CD4^+^ and CD8^+^ T cells were quantified using flow cytometry. Vaccination with rS1 in conjunction with FliC resulted in higher frequencies of IFN‐γ^+^ and IL‐4^+^ CD4^+^ T cells, as well as increased expression of IL‐17A^+^ CD4^+^ T cells, compared to the rS1 group. Conversely, in the rS1 + Alum group, only IL‐4^+^ CD4^+^ T cells were significantly elevated relative to the rS1 group (Figure 5a and Figure S9a). In the rS1 + FliC group, a significantly higher proportion of antigen‐specific IFN‐γ^+^ CD8^+^ T cells was observed compared to the rS1 group. No significant differences were noted in the proportions of IL‐4^+^ and IL‐17A^+^ CD8^+^ T cells between the groups (Figure 5b and Figure S9b).

## Discussion

Fewer COVID‐19‐related hospitalisations and mortalities were observed in countries with higher vaccination coverage, indicating that vaccines can reduce disease severity and mitigate the effects of the infection (Dhama *et al*., 2023). WHO statistical data indicates that, as of 31 December 2023, the countries with the highest numbers of individuals vaccinated against COVID‐19 are the United States, Brazil, and Japan (World Health Organization, 2023). Vaccination rates in some developing regions remain low, highlighting the critical need for the continued development of affordable COVID‐19 vaccines that are easy to transport and store to meet the global demand.

Plant molecular farming offers an alternative to conventional production platforms, particularly for developing countries (Ahmad *et al*., 2012). Rice is among the most important food crops globally. Therefore, this study aims to investigate low‐cost and efficient recombinant antigens in rice. The results showed that the rS1 protein was successfully stably expressed in the pGt1::S1 transgenic rice seeds, as confirmed through mass spectrometry. Various types of recombinant proteins can accumulate highly stably and in high amounts in the endosperm (Peters and Stoger, 2011), providing an ideal environment for the storage of the recombinant rS1 protein. However, the rS1 protein was not expressed in the pActin::S1 transgenic rice, indicating that the design strategy of pGt1::S1 is superior. The Gt1 signal peptide sequence enhanced the expression and accumulation of rS1 proteins in transgenic rice. This sequence is a key factor in improving the expression of target proteins in plant bioreactors, specifically through the application of receptor plant‐derived gene expression regulatory elements and their targeting sequences.

Glycosylation modifications of plant‐based vaccines have been demonstrated to significantly enhance immune responses (Arcalis *et al*., 2013). The SARS‐CoV‐2 S protein contains multiple glycosylation sites (Shajahan *et al*., 2020). The results demonstrated that the molecular weight of rS1 was consistent with the expected size after PNGase F treatment, indicating that N‐glycosylation modification occurred during the expression of the S1 protein in rice endosperm. In the investigation of the SARS‐CoV‐2 trimer subunit vaccine candidate, the S1 protein expressed in Chinese hamster ovary (CHO) cells exhibited a molecular weight of 116 kDa before deglycosylation (Liang *et al*., 2021), consistent with the current findings. While glycosylation modification is recognized for maintaining conformational stability and high antigenicity, concerns persist regarding the potential for plant‐derived glycans to cause allergies. The HA‐based vaccine developed by Medicago has been reported to be safe, even among volunteers with pre‐existing plant allergies, suggesting that these concerns are largely unfounded (Ward *et al*., 2014).

A previous study has demonstrated that the SARS‐CoV‐2 RBD protein is expressed rapidly in tobacco, achieving an expression level of 25 μg/g fresh weight (Siriwattananon *et al*., 2021). In contrast to the transient expression system, the stable expression system can integrate foreign genes into the genome, transmit the target genes to subsequent generations and facilitate the rapid expansion of antigen protein production (Gelvin, 2010). In this study, six homozygous transgenic rice lines exhibiting stable rS1 protein expression were obtained. The rS1 protein expression levels in rice seeds ranged from 96 μg/g to 282 μg/g of dry weight, higher than those reported in previous studies. For example, the expression level of the RBD in pUbi‐RBD rice seeds reached 5.31 μg/g of dry weight (Saba‐Mayoral *et al*., 2023). The expression level of a chimeric fusion protein comprising the *Ascaris suum* protective antigen As16 and the cholera toxin B subunit (CTB) in the rice endosperm was 50 μg/g of seed (Matsumoto *et al*., 2009). The rS1 protein constituted up to 6.3% of the total seed protein. Various studies have reported protein production in rice with yields ranging from 0.678% to 4.521% for VP2 protein (Wu *et al*., 2007) and up to 15% for the Cry j 1 antigen (Yang *et al*., 2007). Variations in expression levels may be attributed to differences in construction strategies, promoter selection and rice varieties (Qu and Takaiwa, 2004). Boehm *et al*. (2000) demonstrated that the endoplasmic reticulum‐specific signal peptide sequence HDEL from bean actin could enhance the transcription and expression of *E. coli* Coenzyme Q in transgenic tobacco, utilizing a similar strategy. Additionally, rS1 transgenic rice seeds exhibited no significant differences in grain size and TGW compared to WT seeds, indicating that the rS1 gene insertion does not affect the agronomic traits of rice seeds, given that grain shape is a significant determinant of rice yield and quality (Tada *et al*., 2003). As the primary storage protein in rice seeds, glutelin constitutes approximately 60–80% of total endosperm protein. Low‐storage protein mutants offer greater capacity for accumulating foreign gene products than standard host plants (Yan *et al*., 2014), facilitating higher levels of foreign gene product accumulation. Therefore, hybridizing S1 transgenic rice with low‐glutelin rice could further enhance rS1 protein expression in future applications.

Subunit vaccines offer a higher safety profile than other vaccine types, and the inclusion of adjuvants can enhance the immune response to the antigen (Liang *et al*., 2020). In this study, the immunogenicity of the purified rS1 protein was evaluated with Alum or flagellin as adjuvants. Alum adjuvants remain the most commonly used approach despite being first discovered nearly 100 years ago (Laera *et al*., 2023). Due to their limited ability to induce robust cell‐mediated (Th1) immune responses, a candidate FliC adjuvant that has been extensively studied in vaccines against multiple pathogens was selected (Cui *et al*., 2018; Hedayat *et al*., 2012). The use of aluminium adjuvants in combination with protein antigens generally induces an enhanced Th2 immune response in mice (Hogenesch, 2013). Similarly, the findings revealed that mice receiving the Alum‐adjuvant rS1 vaccine exhibited increased serum IgG and IgG1 antibodies alongside high levels of IL‐4 and IL‐10 production and an increased percentage of IL‐4^+^ CD4^+^ T cells. These findings are consistent with previous observations in Alum‐adjuvanted vaccines for respiratory diseases in SARS‐CoV (Honda‐Okubo *et al*., 2015). In this study, mice in the rS1 + FliC group exhibited a significant increase in IgG1 and IgG2a levels compared to those in the rS1 group. Additionally, the FliC‐adjuvant rS1 vaccine elicited higher proportions of antigen‐specific IFN‐γ^+^ and IL‐4^+^ CD4^+^ T cells, as well as IL‐17^+^ CD4^+^ T cells, in mouse splenocytes, indicating that FliC promotes a mixed Th1/Th2/Th17 immune response. Previous studies have documented an elevation in IL‐17 levels subsequent to flagellin injection (Habibi *et al*., 2018; Lai *et al*., 2015). Bacterial flagellin is recognized for its ability to activate the inflammasome through the NOD‐like receptor family (NLRC4) and TLR5, thereby inducing a mixed Th1/Th2/Th17 immune response and consequently increasing the concentrations of IFN‐γ, IL‐4 and IL‐17 cytokines (Bakht Azad *et al*., 2018; Gachpazan *et al*., 2025; Hajam *et al*., 2017). These observations are consistent with our findings.

Cellular and humoral immune responses induced by vaccines are both crucial for preventing and controlling viral infections (Maharjan and Choe, 2021). To further evaluate the capacity of the rS1 vaccine to induce a cellular immune response, mouse splenocytes were collected after the third immunization, and the number of splenic lymphocytes secreting IFN‐γ and IL‐4 was analysed using an ELISpot assay. The rS1 protein alone induced significant IFN‐γ and IL‐4 expression compared to the control. The addition of an Alum‐adjuvant did not significantly increase the number of IFN‐γ secreting cells, consistent with the findings from a study on a tobacco‐expressed RBD subunit vaccine, where the number of IFN‐γ‐secreting T cells in the Alum‐adjuvant immunization group did not significantly increase compared to the RBD‐alone group (Siriwattananon *et al*., 2021). In contrast, the addition of flagellin significantly increased the number of IFN‐γ‐secreting cells. These results suggest that plant‐produced rS1 could induce both humoral and cellular immune responses.

The RBD of the S1 protein forms crucial interactions with ACE2 receptors in humans for facilitating viral entry (Watanabe *et al*., 2020). In this study, the rS1 protein demonstrated a high affinity for the ACE2 protein produced by mammalian cells. At elevated ACE2 concentrations, glycosylated rS1 demonstrated stronger binding affinity to ACE2 compared to deglycosylated rS1, akin to the S1 protein produced in HEK293 cells. This was corroborated by studies indicating that deglycosylation of the HEK293‐produced spike protein diminished its binding capability to ACE2 (Bejoy *et al*., 2023). Previous studies have shown that non‐glycosylated spike protein derived from chloroplasts and glycosylated spike protein from rice both exhibit high affinity for ACE2 (Saba‐Mayoral *et al*., 2023; Singh *et al*., 2023), whereas non‐glycosylated spike protein from *E. coli* does not bind effectively to ACE2 (Bejoy *et al*., 2023). Therefore, further investigation is warranted to elucidate the role of glycosylation in the binding of the RBD to ACE2 during SARS‐CoV‐2 infection, as this affinity may be directly or indirectly influenced by glycosylation. Furthermore, the results revealed that the FliC and Alum‐adjuvanted rS1 vaccine induced significantly higher neutralizing antibodies against the SARS‐CoV‐2 pseudovirus than in the control group. A similar result was observed in the serum of mice immunized with the tobacco‐derived RBD‐Fc fusion protein (Shanmugaraj *et al*., 2022), indicating the potential of rice‐produced S1 subunit vaccine candidate as an effective SARS‐CoV‐2 vaccine.

Several studies have shown that T cell immunity induced by existing vaccines is highly conserved across SARS‐CoV‐2 variants (Mazzoni *et al*., 2022; Naranbhai *et al*., 2022), suggesting that the development of second‐generation COVID‐19 vaccines could effectively harness the role of T cells. This approach may include adding other conserved antigens, such as N and M proteins, along with the inclusion of a potent adjuvant to support the creation of broad‐spectrum SARS‐CoV‐2 vaccines. The antigen‐flagellin fusion expression strategy enables a close association between the antigen and flagellin while maintaining their relatively independent spatial structures and biological activities. This approach facilitates proper protein folding and optimizes antigen epitope presentation (Honko *et al*., 2006). Its adjuvant effect is superior to that of immunization with a flagellin and antigen mixture (Mizel and Bates, 2010). Therefore, future studies could explore the use of antigen‐flagellin expression and T‐cell epitope‐based vaccine development strategies for creating cost‐effective COVID‐19 vaccines produced in rice bioreactors.

Additionally, studies have shown that the cell wall of rice seeds can serve as a natural ‘biological capsule’, allowing antigens stored in seeds to resist hydrolysis by gastrointestinal proteases during oral immunization (Takagi *et al*., 2010). The development of rice seed‐based biopharmaceuticals and edible vaccines holds significant potential for widespread applications. Future studies will focus on a detailed investigation of oral immunization. While edible, plant‐based therapeutics remain predominantly at the preclinical stage of development, successful advancements in this field could establish new classes of pharmaceutical products. Current production systems are expected to continue dominating vaccine manufacturing; however, with advancements in production and technology, the balance may gradually shift toward molecular agriculture.

## Conclusion

This study demonstrates that the rice‐derived S1 glycoprotein of SARS‐CoV‐2 remains stable and highly expressed in transgenic rice seeds. Furthermore, the rS1 protein exhibits a high affinity for the human ACE2 protein and elicits both humoral and cellular immune responses in mice. These results suggest the potential of using transgenic rice‐based expression systems as bioreactors for future vaccine production, particularly in developing countries. This strategy represents a unique and effective platform to produce safe, efficient and cost‐effective subunit vaccines for COVID‐19 and other viruses.

## Materials and methods

### Plant materials and cultivation conditions

The rice variety ‘Zhonghua11’ (ZH11), a model known for its high transformation efficiency, was used for generating S1 transgenic lines. All rice materials were grown in paddy fields at Yangzhou University or Lingshui County, Hainan Province under the same climatic conditions and crop management practices.

### Mice and ethical considerations

Specific pathogen‐free BALB/c female mice, aged 6–8 weeks, were obtained from Beijing Vital River Laboratory Animal Technology (Beijing, China). The mice were housed under a 12 h light/darkness cycle at 25 °C and 50% humidity. All mice were provided ad libitum access to standard chow diets during the assays. All mouse experiments were approved by the Ethics Committee of the Animal Experiments of Yangzhou University [Approval ID: SYXK (Su) 2022‐0044] and conducted according to the guidelines for animal care and ethics.

### Construction of plant vector and rice genetic transformation

The DNA sequence encoding the SARS‐CoV‐2 S1 gene (GenBank accession No. NC_045512.2) was synthesized with rice codon bias by Genscript Biotechnology Inc (Nanjing, China) (Figure S1). The S1 gene was amplified by polymerase chain reaction (PCR) using primers Gt1‐S1F and Gt1‐S1R (Table S1), and cloned into the plant binary vector pCAMBIA1300Gt1 (pGt1) after digestion with *Bam*H I and *Sac* I, incorporating the rice glutelin (Gt1) promoter. Additionally, the S1 gene was amplified using PCR with the primers Actin‐S1F1/2 and Actin‐S1R (Table S1) and cloned into the vector pCAMBIA1300Actin (pActin) digested by *Xba* I and *Sal* I, which contains the constitutive promoter (Actin). The Kozak sequence and a sequence encoding the endoplasmic reticulum retention signal ‘KDEL’ were fused to the S1 gene to enhance its accumulation levels. Furthermore, the Gt1 signal peptide was added to the N‐terminal of the codon‐optimized S1 gene for the Gt1 promoter vector (Figure 1a). The recombinant plasmids pGt1‐S1 and pActin‐S1 were identified and transformed into the callus derived from rice cultivar ZH11 through *Agrobacterium‐*mediated transformation as previously described (Slamet‐Loedin *et al*., 2014) (Figure S2a).

### Screening for positive transgenic plants

PCR analysis: Total genomic DNA was extracted from young transgenic rice leaves (T_1_‐T_3_ generation) using the CTAB method (Doyle and Doyle, 1987). Positive transgenic rice plants were identified through PCR analysis using two sets of primers, S1‐F1/S1‐R1 (for T_1_ generation) and S1‐F2/S1‐R2 (for T_2_ and T_3_ generation) (Table S1). The PCR program included one cycle at 95 °C for 5 min, followed by 35 cycles of 95 °C for 30 s, 54 °C for 30 s and 72 °C for 45 s (2 min 30 s for T_2_ and T_3_ generation), concluding with a 72 °C incubation for 10 min. The PCR products were identified by 1% agarose gel electrophoresis.

Hygromycin resistance analysis: Seeds (*n* = 50 per line) harvested from T_3_ plants were subjected to hygromycin resistance screening, with non‐transgenic (WT) and heterozygous (T_1_‐8) lines serving as controls. Initially, the seeds were germinated in sterile water under dark conditions. Subsequently, the germinated seedlings were transferred to sterile water supplemented with 25 mg/L hygromycin and exposed to light. After a period of 7 days, the proportion of hygromycin‐resistant plants (H^R^) was determined.

### Total RNA extraction and probe‐based quantitative reverse transcription polymerase chain reaction analysis

Developing seed samples (T_3_ generation) were collected 15 days after flowering (DAF) to analyse S1 gene expression. Total RNA was extracted from the collected rice samples using the FastPure Plant Total RNA Isolation Kit (Vazyme, Nanjing, China), according to the protocol of the manufacturer. Complementary DNA (cDNA) was synthesized from mRNA using a Hiscript III RT Super mix for qPCR Kit (Vazyme). Primers (qS1‐F/qS1‐R) and TaqMan probes (qS1‐P) for quantitative reverse transcription polymerase chain reaction (qRT‐PCR) were designed based on the S1 gene sequence to amplify and detect a 200‐base pair segment of the S1 gene (Table S1). The plasmid pGt1‐S1 was diluted 10‐fold (10^1^–10^8^ copies/μL) and used as a template for the qRT‐PCR standard. All samples, including standard template and negative controls, were added to a 96‐well plate with three replicates set up. The reaction was performed on an ABI 7500 (Applied Biosystems, USA) with the following steps: 95 °C for 30 s, followed by 40 cycles of 95 °C for 5 s and 60 °C for 34 s. After the reaction, a standard curve was generated from the fluorescence curve, and Ct values were analysed via the system. The copy numbers of the S1 gene were quantified using the following equation: copies/μL = [(6.02 × 10^23^ copies/mol) × concentration of plasmid g/μL]/(number of base pairs of the plasmid × 2 × 324.5).

### Extraction of total protein from rice seeds and leaves

Mature rice seeds (T_1_–T_3_ generation) were air‐dried and polished using a grain polisher (Shuanglong, Korea). The seeds were ground into powder, and total protein was extracted with extraction buffer (125 mmol/L Tris–HCl, 4 mol/L Urea, 4% SDS, 5% beta‐mercaptoethanol, pH6.8) in a 1:5 (w/v) ratio at room temperature for 3 h with constant stirring. The crude extract was clarified by centrifugation at 12 000 rpm for 20 min at 4 °C, and the supernatant was collected. Rice leaves (T_1_ generation pActin::S1 transgenic rice) were cut and ground into powder in liquid nitrogen, and total protein was extracted using extraction buffer (50 mmol/L Tris–HCl, 150 mmol/L NaCl, 0.5% SDS, pH 7.5) at 4 °C for 3 h with constant stirring. The crude extract was clarified by centrifugation at 12 000 rpm for 10 min at 4 °C, and the supernatant was collected. The extracted protein samples were verified using Western blot, with the non‐transgenic rice strain ZH11 as a negative control.

### Deglycosylation

Deglycosylation of the rS1 protein was assessed by treatment with PNGase F (Novoprotein, Suzhou) according to the instructions of the manufacturer. Briefly, total protein from rice seeds was mixed with glycoprotein denaturing buffer and incubated at 100 °C for 10 min. The mixture was then added and combined with the enzyme digestion system —2 μL of 10 × PNGase F reaction buffer, 2 μL 10% NP‐40, 5 μL PNGase F (50 U/μL) and 5 μL ddH_2_O. The enzyme‐digestion reaction was performed at 37 °C for 90 min, followed by thermal inactivation at 75 °C for 10 min. The deglycosylated protein samples were analysed by Western blotting.

### Western blot analysis

Protein samples were separated using 12% sodium dodecyl sulphate–polyacrylamide gel electrophoresis (SDS‐PAGE) and then transferred to a nitrocellulose membrane (Millipore, Darmstadt, Germany). The membrane was blocked with 5% skim milk in phosphate‐buffered saline with tween‐20 (PBST) buffer for 2 h and washed 3 times with PBST. It was then incubated with rabbit polyclonal antibody against RBD protein (Sino Biological, Beijing, China) or rabbit anti‐Flag monoclonal antibody (Cell Signalling Technology) at a 1:1000 dilution. Mouse anti‐Hsp82 monoclonal antibody (1:10 000) was employed to detect rice heat shock protein (Hsp) as an internal control for normalizing gene expression. Subsequently, it was reacted with monoclonal goat anti‐rabbit IgG‐horseradish peroxidase (HRP) (Cell Signalling Technology) at a 1:5000 dilution. Finally, West Pico PLUS chemiluminescent substrate (Solabao, Beijing) was added to react for 10 min, and images were captured using an Amersham Imager 600 imager (General Electric, USA). Band intensity was analysed with Image‐J and normalized via the Hsp82 reference.

### Mass spectrometry analysis of rS1 protein

Total protein was extracted from T_2_ generation rice seeds and separated by SDS‐PAGE. The target protein was cut and digested with trypsin to obtain fragmented peptide segments. The sample was analysed by Liquid Chromatography Tandem Mass Spectrometry (LC–MS/MS) at Zhongke New Life Co., Ltd (Shanghai, China). The detected peptide sequence was aligned with the S1 amino acid sequence to determine peptide coverage.

### rS1 protein quantification

Total protein was extracted from T_3_ generation rice seeds, and S1 protein concentration in the extract was measured through quantitative Western blot assay (Lallier *et al*., 2019). Standard S1 protein (Sino Biological) was briefly diluted in a 2‐fold gradient to 20, 10, 5 and 2.5 μg/mL, with non‐transgenic plant ZH11 extract (wild type; WT) as a negative control. Both rice extract and S1 protein standard were analysed by Western blot using rabbit polyclonal antibody against RBD protein (Sino Biological). A standard curve was generated from the grey values and concentrations of the standard S1 protein, enabling the determination of S1 protein content in seeds. Band intensity was analysed via Image‐J. Additionally, total protein content was assessed using the Enhanced BCA Protein Assay Kit (Beyotime, Shanghai, China), and the percentage of rS1 protein within the total protein was calculated.

### Rice size and thousand‐grain weight analysis

For agronomic trait analysis, 10 mature seeds were randomly selected from the T_3_ generation, harvested, air‐dried and assessed for Grain length (GL), grain width (GW), grain length‐width ratio (GL/GW) and thousand‐grain weight (TGW).

### Purification of rS1 protein

Total protein was extracted from the T_3_ generation rice seeds using an extraction buffer (50 mmol/L Tris, 10 mmol/L NaCl, pH 9.5). The supernatant was aspirated and passed through a 0.45 to −0.22 μm filter (Millipore). Subsequently, the resulting extract was loaded onto a HITRAP Q FF purification column (Cytiva) with buffer A (50 mmol/L Tris, pH 9.5). Impurities were removed using 10% and 100% buffer B (50 mmol/L Tris, 1 mol/L NaCl, pH 9.5). The rS1 protein was eluted with 20%, 30%, 50% and 70% buffer B. Subsequently, the enriched protein sample was loaded onto a Sephacryl S‐200 HR gel filtration purification column (Cytiva) while the different peaks were collected. The purity of the S1 protein was confirmed using SDS‐PAGE and Western blot analysis.

### ACE2 protein binding activity

The purified rS1 protein was diluted to a concentration of 1 μg/mL using carbonate buffer and coated onto 96‐well microplates overnight at 4 °C. The deglycosylated rS1 protein, S1 protein expressed in HEK293 cells (Sino Biological), and phosphate‐buffered saline (PBS) served as controls. After three washes with PBST, the plate was blocked with 1% serum albumin (BSA) in PBS at 37 °C for 2 h. After an additional three washes, ACE2 protein with His tag (Absin, Shanghai) was diluted to 100 μg/mL using a two‐fold gradient dilution and incubated at 37 °C for 2 h. Subsequently, an HRP‐labelled mouse anti‐His antibody (1:5000) was added, and the reaction was conducted at 37 °C for 1 h. After five washes, Tetramethylbenzidine (TMB) substrate (Solabao) was added to each well and incubated for 10 min, after which the optical density at 450 nm (OD_450_) was measured using a microplate reader (Bio‐Tek).

### Extraction and identification of the *Salmonella* FliC adjuvant

The *S*. Typhimurium strain ATCC14028s (pTrc99a‐*fliC*‐WT) was cultured in the presence of 100 μg/mL ampicillin. Subsequently, highly purified FliC was isolated according to the previously described method (Song *et al*., 2023). The extracted FliC protein was analysed using SDS‐PAGE and Western blot analysis with anti‐FliC antibodies. The protein was treated with the ProteoSpin™ Endotoxin Removal Kit Maxi (Norgen Biotek Corp., Thorold, Canada) to eliminate endotoxins. Residual endotoxin levels were measured using the Chromogenic Endpoint Tachypleus Amebocyte Lysate (CE TAL) Assay Kit (Chinese Horseshoe Crab Reagent Manufactory Co., Xiamen, China) until the final concentration reached <0.05 EU/μg.

### Immunization and sampling procedures

Female BALB/c mice, aged 6–8 weeks, were randomly allocated into four groups (*n* = 6). The mice were immunized intramuscularly at weeks 0, 2 and 4 with 20 μg of rS1 protein, 20 μg of rS1 protein mixed with 20 μg of FliC adjuvant (rS1 + FliC), 20 μg of rS1 protein mixed 1:1 (w/v) with aluminium adjuvant (ThermoFisher, USA) (rS1 + Alum) or PBS (100 μL) as a control. Serum samples were collected on day 12 after the second and third immunizations to analyse antibody immune responses. Two weeks after the last vaccination, spleens were harvested from the mice to assess cellular immune responses.

### Enzyme‐linked immunosorbent assay

The S1‐specific serum IgG and IgG subclasses (IgG1 and IgG2) were evaluated using enzyme‐linked immunosorbent assay (ELISA). Briefly, the purified rS1 protein was diluted to a concentration of 1 μg/mL in carbonate buffer (pH 9.6) and coated onto 96‐well microplates overnight at 4 °C. Following three washes with PBST, the plates were blocked with 1% BSA for 2 h at 37 °C. The serum samples from the different vaccine groups were serially diluted and added to the plate. After a 2‐h incubation at 37 °C, HRP‐labelled anti‐mouse IgG (1:5000), IgG1 (1:3000) and IgG2 (1:3000) antibodies were introduced. Subsequently, the plate was incubated at 37 °C for 1 h. After five washes, TMB substrate was added to each well. After three washes with PBST, the OD_450_ was measured using a microplate reader (BioTek, Winooski, VT, USA). Antibody titres were defined as the reciprocal of the highest sample dilution with an absorbance reading above the cut‐off value.

### Pseudovirus neutralization assay

The neutralizing antibody (NAb) level in mouse serum was measured using the SARS‐CoV‐2 pseudovirus neutralization antibody detection kit (WT) with a luciferase (Luc) reporter (GenScript, Nanjing, China), following the instructions of the manufacturer. Briefly, 50 μL of SARS‐CoV‐2 pseudovirus was mixed with an equal volume of two‐fold serially diluted serum samples and a positive neutralizing antibody (ACE2‐Fc) in a 96‐well plate. Both the virus and the samples were diluted in Opti‐MEM. After incubation at room temperature for 1 h, the virus‐serum mixtures were sequentially added to 96‐well cell plates. Subsequently, 50 μL per well of an Opti‐HEK293/ACE2 cell suspension was introduced and incubated at 37 °C in a CO_2_ incubator. After 24 h, 50 μL of Luc reagent (Promega, Madison, WI, USA) was added, and the plate was incubated at room temperature for 3–5 min. Subsequently, the absorbance at 450 nm was measured using a microplate reader (BioTek). The NAb titres were defined as the reciprocal serum dilution at which the relative light units (RLU) were reduced by 50% compared to the RLU in the virus‐control wells after subtracting the background RLU measured in the cell‐control wells.

### Isolation of splenocytes from immunized mice

Two weeks after the last vaccination, spleens were harvested from the mice to assess cellular immune responses. Splenocytes were isolated from the immunized mice, following previously described methods (Song *et al*., 2017). For cytokine detection, splenocytes were plated in 24‐well plates (1 × 10^6^ cells per well in 0.5 mL) of complete RPMI 1640 medium supplemented with 10% fetal bovine serum and 1% penicillin–streptomycin (Gibco). The cells were stimulated with 5 μg/mL rS1 protein. After 5 h, the cells were harvested separately for RNA extraction and qRT‐PCR quantification. After 48 h, cytokine secretion levels in the culture supernatants were quantified using ELISA kits for IFN‐γ, IL‐4, IL‐10 and IL‐17A (BD, San Jose, CA, USA), and TNF‐α (BioLegend, San Diego, CA, USA), following the manufacturer's protocols.

### RNA extraction and quantitative reverse transcription polymerase chain reaction quantification of cytokines

Total mRNA was extracted from the splenic lymphocytes of immunized mice using an RNAprep Pure Cell/Bacteria Kit (TIANGEN, Beijing), and cDNA was synthesized from the mRNA using the PrimeScript RT Reagent Kit (Takara, Dalian, China) following the instructions of the manufacturer. The mRNA levels of the cytokines IFN‐γ, IL‐4, TNF‐α, IL‐10, and IL‐17A were assessed using qRT‐PCR analysis with SYBR Green Master Mix (Roche Diagnostics, Tokyo, Japan) on an ABI 7500 Fast Real‐Time PCR System. PCR amplification was performed in a total volume of 20 μL containing 10 μL of 2× SYBR Premix Ex Taq II, 2 μL of diluted cDNA and 0.8 μL of each primer. The qRT‐PCR programme started with a denaturation step at 95 °C for 30 s, followed by 40 cycles of 95 °C for 5 s and 60 °C for 34 s. Relative quantifications of the mRNA of target genes are presented as the comparative threshold cycle (CT) number for each sample ($2^{-\Delta \Delta C_{T}}$). Gene expression was normalized to the corresponding β‐actin level. Table S2 provides the sequences of the primers used for qRT‐PCR, which were synthesized by GenScript Biotech Co., Ltd.

### Cell proliferation assay

Splenic lymphocyte proliferation was measured using the Cell Proliferation ELISA Kit (Roche Diagnostics) following the instructions of the manufacturer. Briefly, splenocyte suspensions (3 × 10^5^ cells per well) were added to 96‐well cell culture plates and stimulated with or without 10 μg/mL of rS1 protein in triplicate, then incubated at 37 °C in a 5% CO_2_ atmosphere for 48 h. Subsequently, 20 μL of BrdU labelling reagent per well was added, followed by incubation for 12 h. The cells were harvested, and the 96‐well plate was dried at 60 °C for 1 h. Intracellular BrdU‐labelled DNA was fixed and denatured at room temperature for 30 min using 200 μL of FixDenat solution per well. After washing, the anti‐BrdU antibody was added and incubated at room temperature for 90 min. Following five washes, TMB substrate was added to each well. The reaction was halted by adding 1 m H_2_SO_4_ solution, and the OD_450_ and OD_690_ were measured using a microplate reader (BioTek). The Stimulation Index (SI) was calculated using the following formula: (OD_450_‐OD_690_) of stimulated wells divided by (OD_450_‐OD_690_) of unstimulated wells.

### Enzyme‐linked immunospot assay

The IFN‐γ and IL‐4 Enzyme‐linked immunospot (ELISpot) kit (BD, USA) was used to detect the number of splenic lymphocytes secreting IFN‐γ or IL‐4, following the manufacturer's instructions Briefly, ELISpot 96‐well plates were coated with anti‐IL‐4 and anti‐IFN‐γ monoclonal capture antibodies and incubated at 4 °C overnight. A suspension of spleen lymphocytes (4 × 10^5^ cells per well) isolated from immunized mice was added. Subsequently, the suspension was stimulated with rS1 protein (5 μg/mL) and incubated at 37 °C in a 5% CO_2_ incubator for 24 h. The plates were washed, and the detection antibodies (biotin‐IFN‐γ and biotin‐IL‐4, diluted 1:250) were added. Following incubation at room temperature for 2 h, streptavidin‐AKP (diluted 1:1000) was added and incubated for 1 h. Finally, BCIP/NBT liquid substrate was added to initiate the reaction. After spots appeared, the reaction was terminated and the number of spots was analysed using an ELISpot reader system scanner (BioSys).

### Flow cytometry

Splenocytes were isolated from vaccinated mice 2 weeks following the final immunization and subsequently restimulated with rS1 protein at a concentration of 10 μg/mL for 6 h at 37 °C in a 5% CO_2_ incubator. This process was conducted in the presence of 10 μg/mL of the protein transport inhibitor Brefeldin A (Solarbio, Beijing, China). Following restimulation, the cells were collected and subjected to surface staining using FITC‐conjugated anti‐mouse CD4 and APC‐conjugated anti‐mouse CD8 antibodies (BioLegend) for 30 min at 4 °C. The cells were then fixed and permeabilized employing the Cyto‐Fast Fix/Perm Buffer Set (BioLegend) as per the manufacturer's protocol. Subsequently, intracellular staining was performed with PE‐conjugated anti‐mouse cytokine antibodies (IFN‐γ, IL‐4 and IL‐17A, BioLegend) for 20 min at room temperature. Finally, the cells were resuspended in PBS and analysed using a FACSAria flow cytometer (BD Biosciences), with data processed via FACSDiva software (BD Biosciences).

### Statistical analysis

All data are expressed as the mean ± standard error of the mean (SEM). Statistical analyses were conducted using GraphPad Prism version 8.0 software, and the data were analysed using Student's *t*‐tests and one‐way analysis of variance (ANOVA). Differences were considered statistically significant at *P < 0.05, **P < 0.01 and ***P < 0.001.

## Author contribution

X.J., Q.Q.L., Z.P. and L.S. conceived and designed the experiments. L.S., Y.W., Y.Z., Y.T., J.W., Y.C., R.T., H.Z. and D.X. performed the experiments. L.S., Y.W., C.M., Y.Z. and F.Q.L. analysed the data. L.S., F.Q.L., Z.P., Q.Q.L. and X.J. wrote, reviewed and edited the manuscript.

## Conflict of interest

The authors declare that they have no conflict of interest.

## Funding

This work was supported by the National Key Research and Development Program of China (2022YFC2604200), the National Natural Science Foundation of China (32102679), the Key research and development program (Modern Agriculture) project of Jiangsu Province (BE2021331), the 111 Project (D18007) and the Priority Academic Program Development of Jiangsu Higher Education Institutions (PAPD).

## Supporting information


**Figure S1** Codon optimisation of S1‐ gene from the SARS‐CoV‐2 Wuhan‐Hu‐1 isolate (NC_045512.2).
**Figure S2** Screening of S1 transgenic plants.
**Figure S3** Hygromycin resistance analysis of transgenic rice.
**Figure S4** Identification of rS1 expression in T_1_ generation transgenic seeds.
**Figure S5** Western blot analysis of rS1 protein in T_1_ generation pActin::S1 transgenic lines.
**Figure S6** Expression level of rS1 protein in T_3_ generation transgenic seeds.
**Figure S7** Effects of rS1 expression on grain size in transgenic rice.
**Figure S8** Preparation and characterisation of *Salmonella* FliC adjuvant.
**Figure S9** Illustration of cytokine‐producing CD4^+^ and CD8^+^ T cells using flow cytometry.

**Table S1** Primers used for construction and identification of S1 gene.
**Table S2** Primer sequences for qRT‐PCR of cytokines.
**Table S3** PCR analysis of genomic DNA from transgenic rice lines.
**Table S4** Hygromycin‐resistant analysis of transgenic rice.

## Data Availability

The data that supports the findings of this study are available in the [Link], [Link] of this article.
