# Supplementary material for: Rice‐derived SARS‐CoV‐2 glycoprotein S1 subunit vaccine elicits humoral and cellular immune responses
Source: Plant Biotechnol J. 2025 Apr 4;23(7):2570–82. doi: 10.1111/pbi.70077 (PMC12205891; doi:10.1111/pbi.70077)
Supplement: Supplementary file 1 — Figure S1 Codon optimisation of S1‐ gene from the SARS‐CoV‐2 Wuhan‐Hu‐1 isolate (NC_045512.2). Figure S2 Screening of S1 transgenic plants. Figure S3 Hygromycin resistance analysis of transgenic rice. Figure S4 Identification of rS1 expression in T1 generation transgenic seeds. Figure S5 Western blot analysis of rS1 protein in T1 generation pActin::S1 transgenic lines. Figure S6 Expression level of rS1 protein in T3 generation transgenic seeds. Figure S7 Effects of rS1 expression on grain size in transgenic rice. Figure S8 Preparation and characterisation of Salmonella FliC adjuvant. Figure S9 Illustration of cytokine‐producing CD4+ and CD8+ T cells using flow cytometry. [file PBI-23-2570-s002.doc]

**Fig. S1**

**
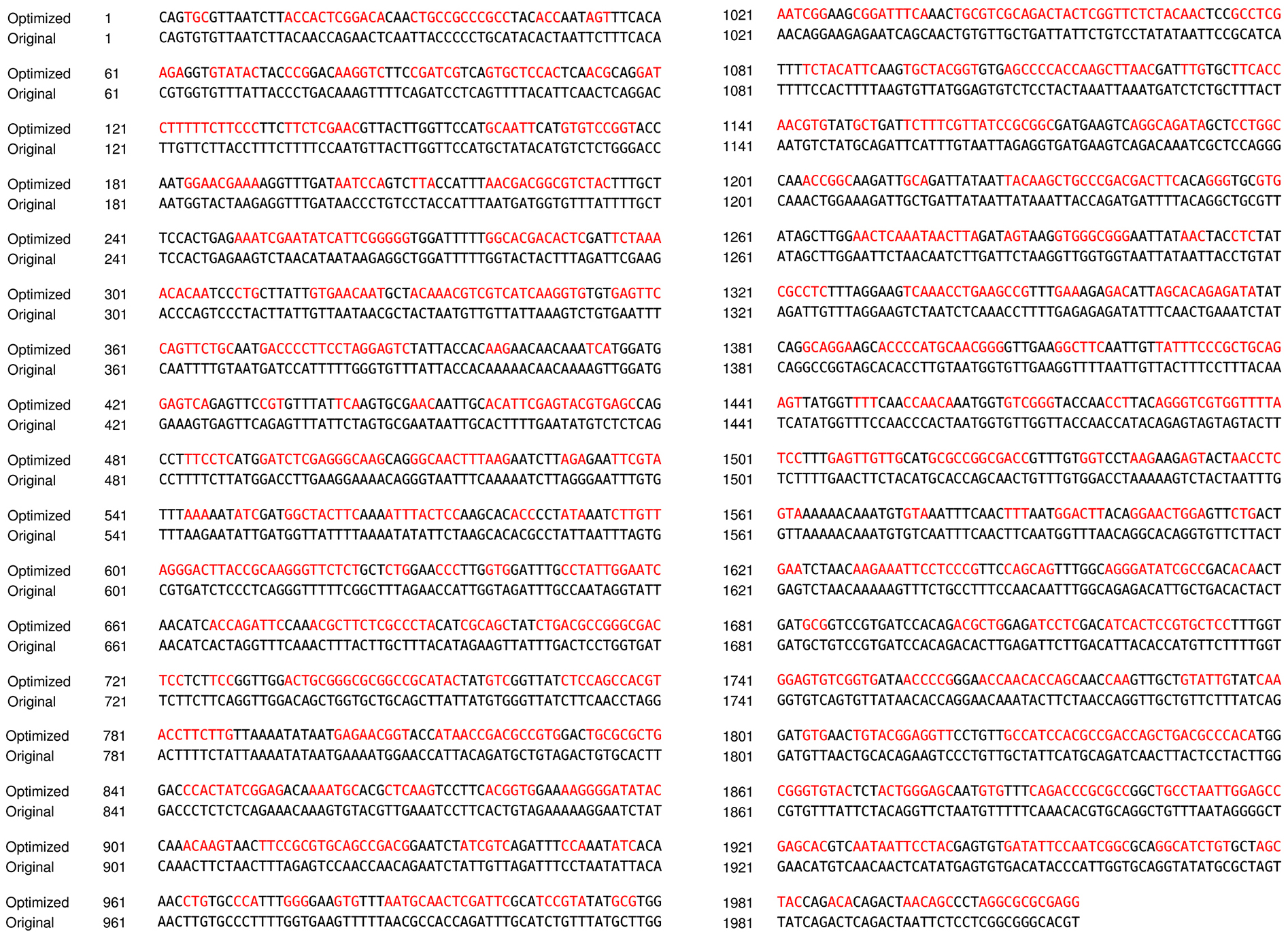
**

**Fig. S1 Codon optimisation of S1- gene from the SARS-CoV-2 Wuhan-Hu-1 isolate (NC_045512.2).** Codon optimisation of the S1 gene is highlighted in red.

**Fig. S2**

**
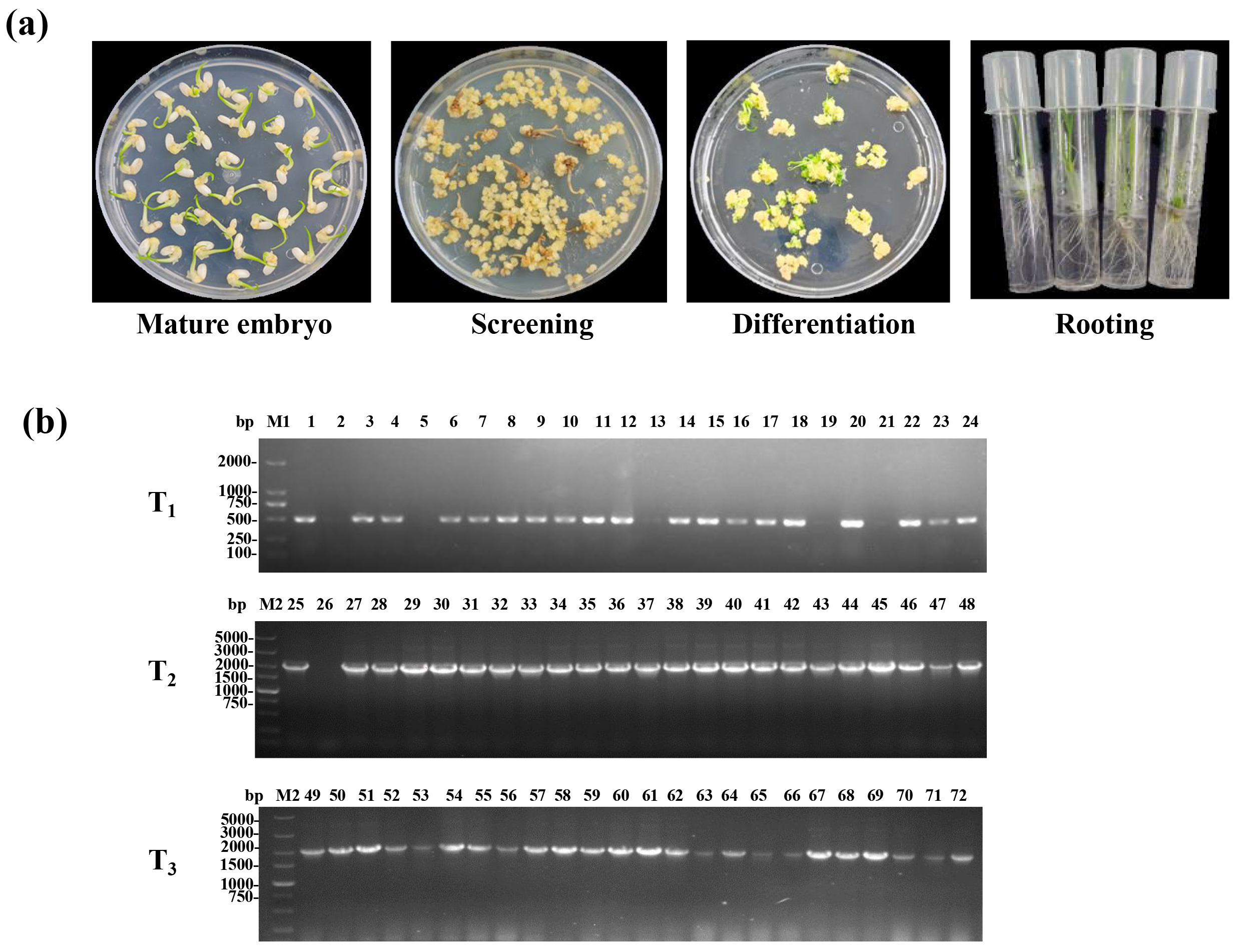
**

**Fig. S2 Screening of S1 transgenic plants. (a)** Rice genetic transformation. Generation of S1 transgenic rice involves the induction of mature embryos, followed by screening, differentiation, and rooting. **(b)** PCR analysis of genomic DNA. Total genomic DNA was extracted from young leaves of pGt1::S1 transgenic rice (T_1_-T_3_ generations). M1: DL2000 DNA marker; M2: DL5000 DNA marker; 1-5: T_1_-10; 6-15: T_1_-11; 16-24: T_1_-12; 25-34: T_2_-1; 35-44: T_2_-2; 45-48: T_2_-3; 49-55: T_3_-4; 56-68: T_3_-5; 69-72: T_3_-6. The results shown were representative of the transgenic rice lines.

**Fig. S3**

**
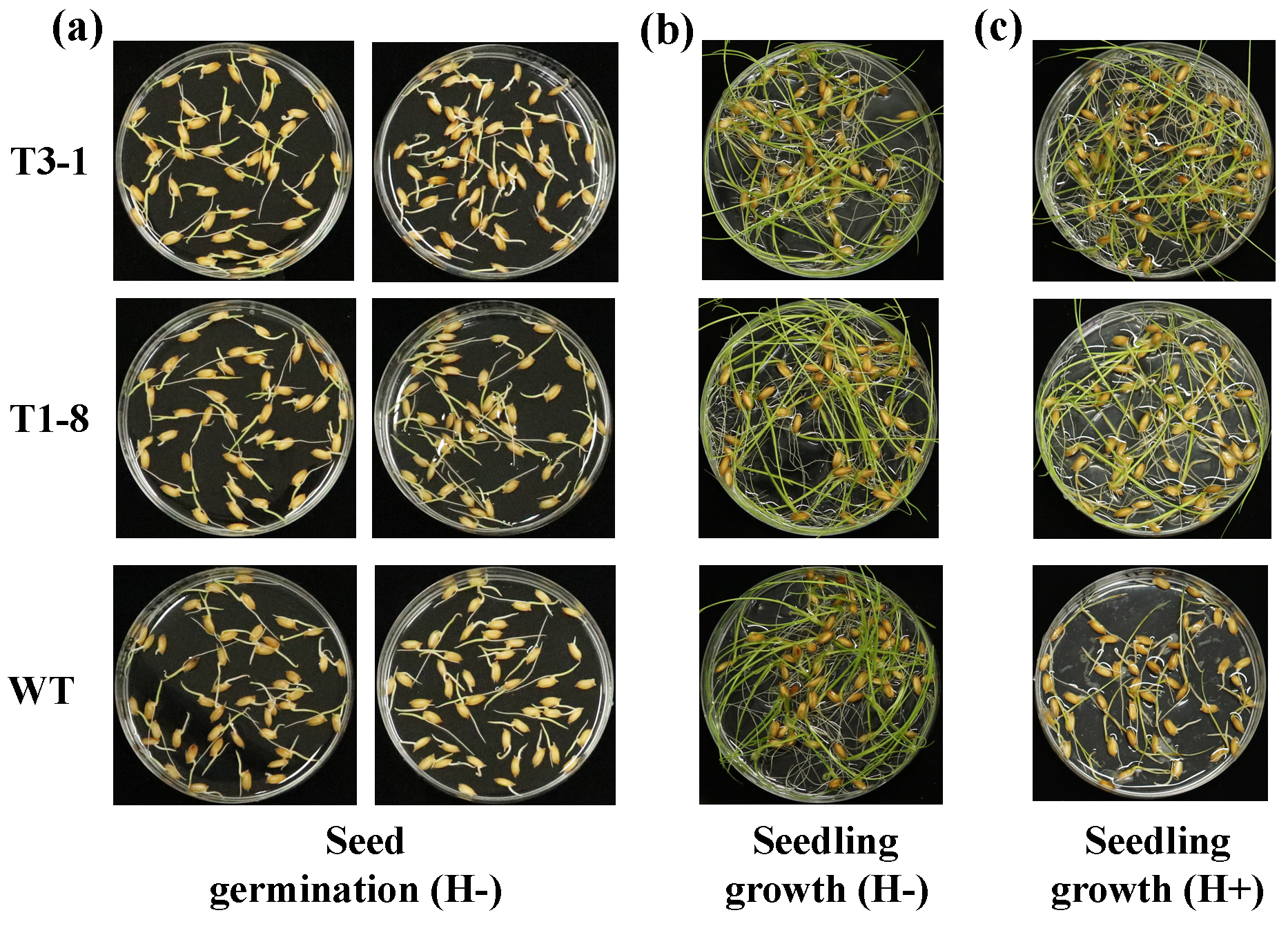
**

**Fig. S3 Hygromycin resistance analysis of transgenic rice.** Seeds (n = 50 per line) collected from T_3_ plants (T_3_-1 to T_3_-6) were screened for hygromycin resistance, with the non-transgenic line (WT) and heterozygous line (T_1_-8) serving as controls. (a) Seed germination. (b) Seedling growth without hygromycin. (c) Seedling growth in the presence of hygromycin (25 mg/L). The results shown are representative of the transgenic rice lines.

**Fig. S4**

**
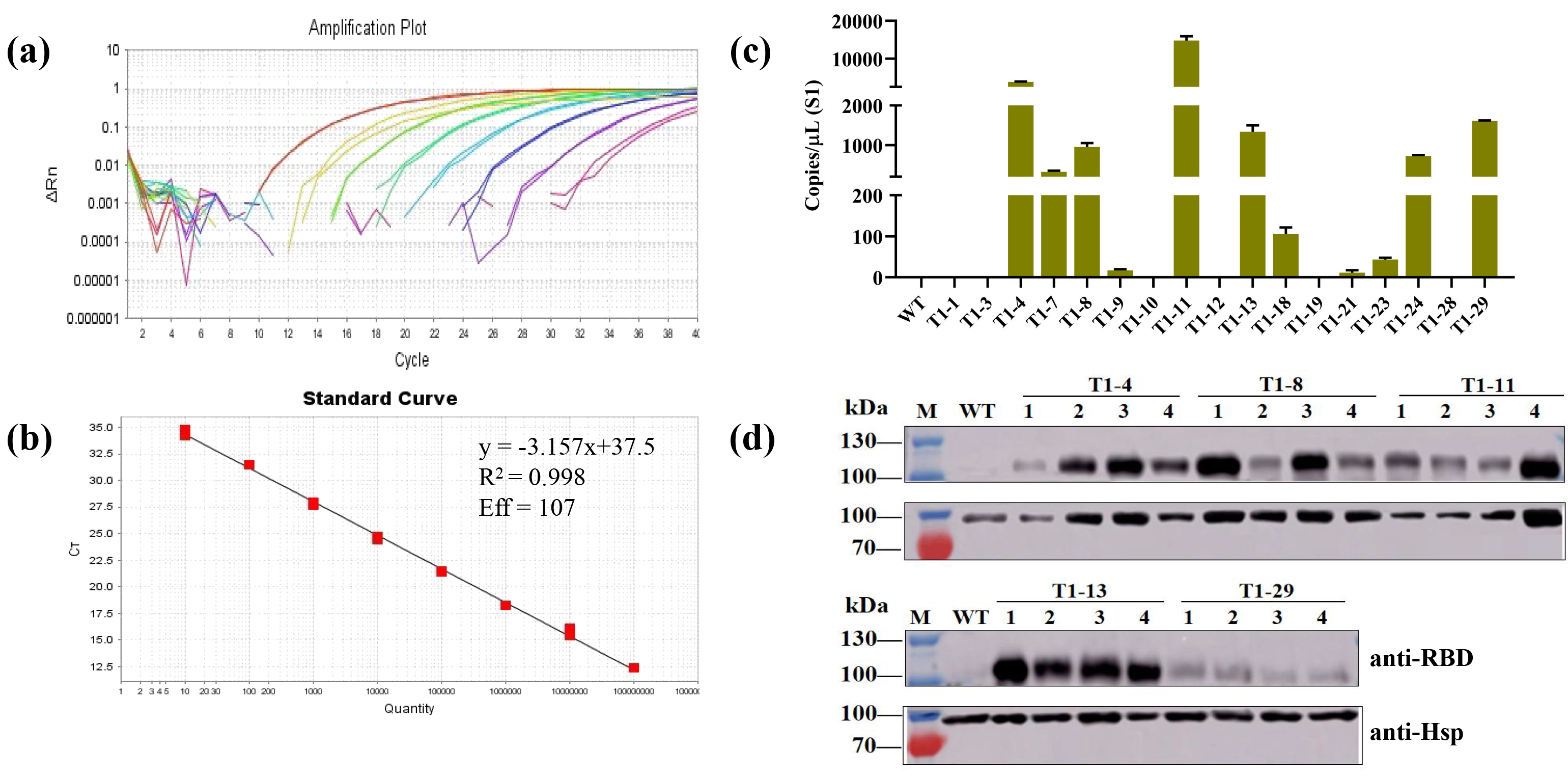
**

**Fig. S4 Identification of rS1 expression in T_1_ generation transgenic seeds.** The mRNA and protein expression levels of rS1 in T_1_ generation pGt1::S1 transgenic seeds and wild-type control (WT) were analysed using qRT-PCR and Western blot analysis, respectively. **(a)** Amplification curve for rS1 detection using qRT-PCR. **(b)** Standard curves for rS1 detection using qRT-PCR. **(c)** mRNA levels of the rS1 gene in different pGt1::S1 transgenic lines and the WT. **(d)** Western blot analysis of rS1 protein in different pGt1::S1 transgenic lines and the WT.

**Fig. S5**

**
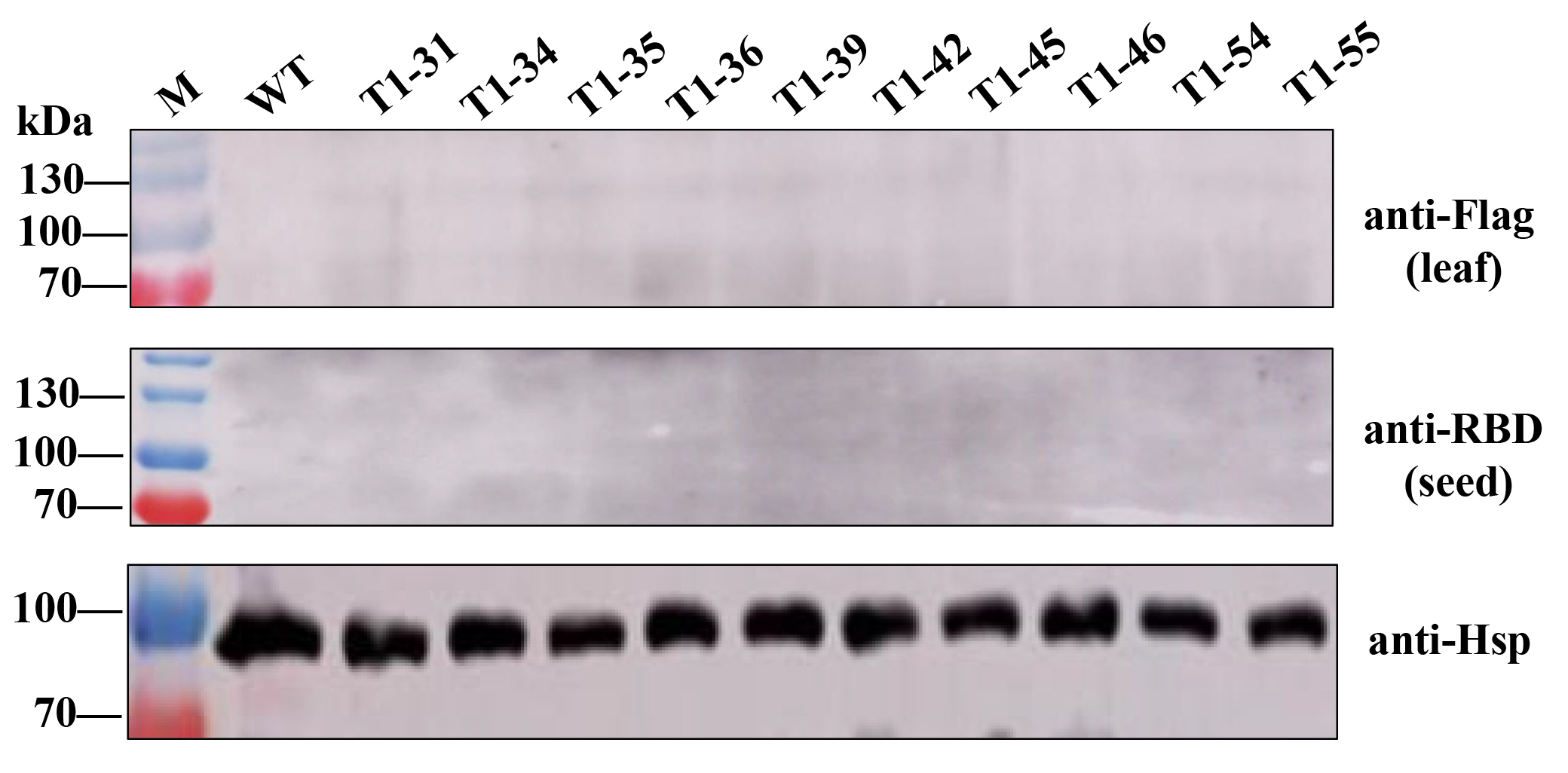
**

**Fig. S5 Western blot analysis of rS1 protein in T_1_ generation pActin::S1 transgenic lines.** Total protein was extracted from T_1_ generation transgenic leaves and seeds for Western blot analysis.

**Fig. S6**

**
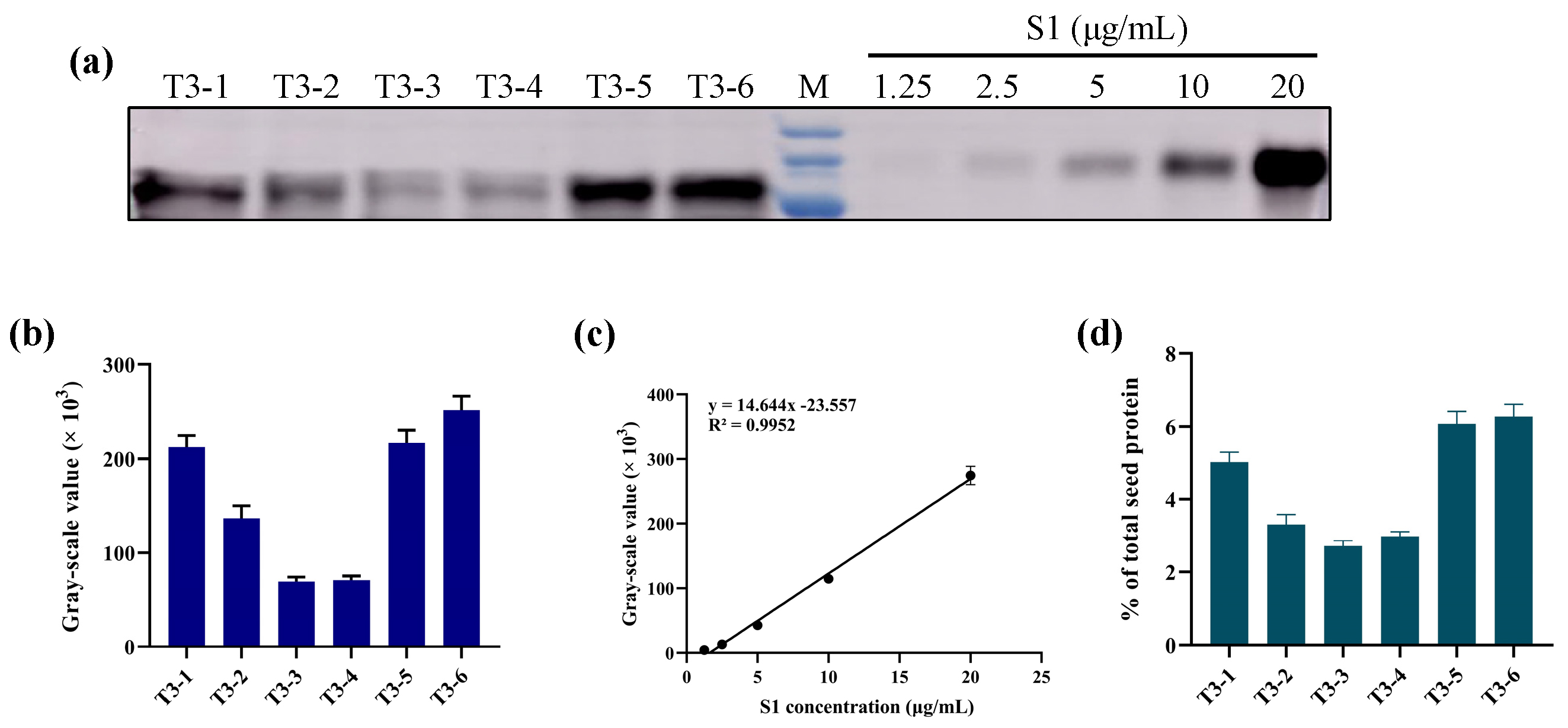
**

**Fig. S6 Expression level of rS1 protein in T_3_ generation transgenic seeds.** Concentration of rS1 protein in T_3_ generation transgenic seeds was analysed using quantitative Western blot analysis. **(a)** Western blot analysis of rS1 standard and total protein extracted from T_3_ generation transgenic rice seeds. **(b)** Quantification of rS1 protein levels in different transgenic plants based on grayscale values. **(c)** Standard curve for detecting rS1 concentration through Western blot analysis. **(d)** The percentage of rS1 protein in the total protein of seeds.

**Fig. S7**

**
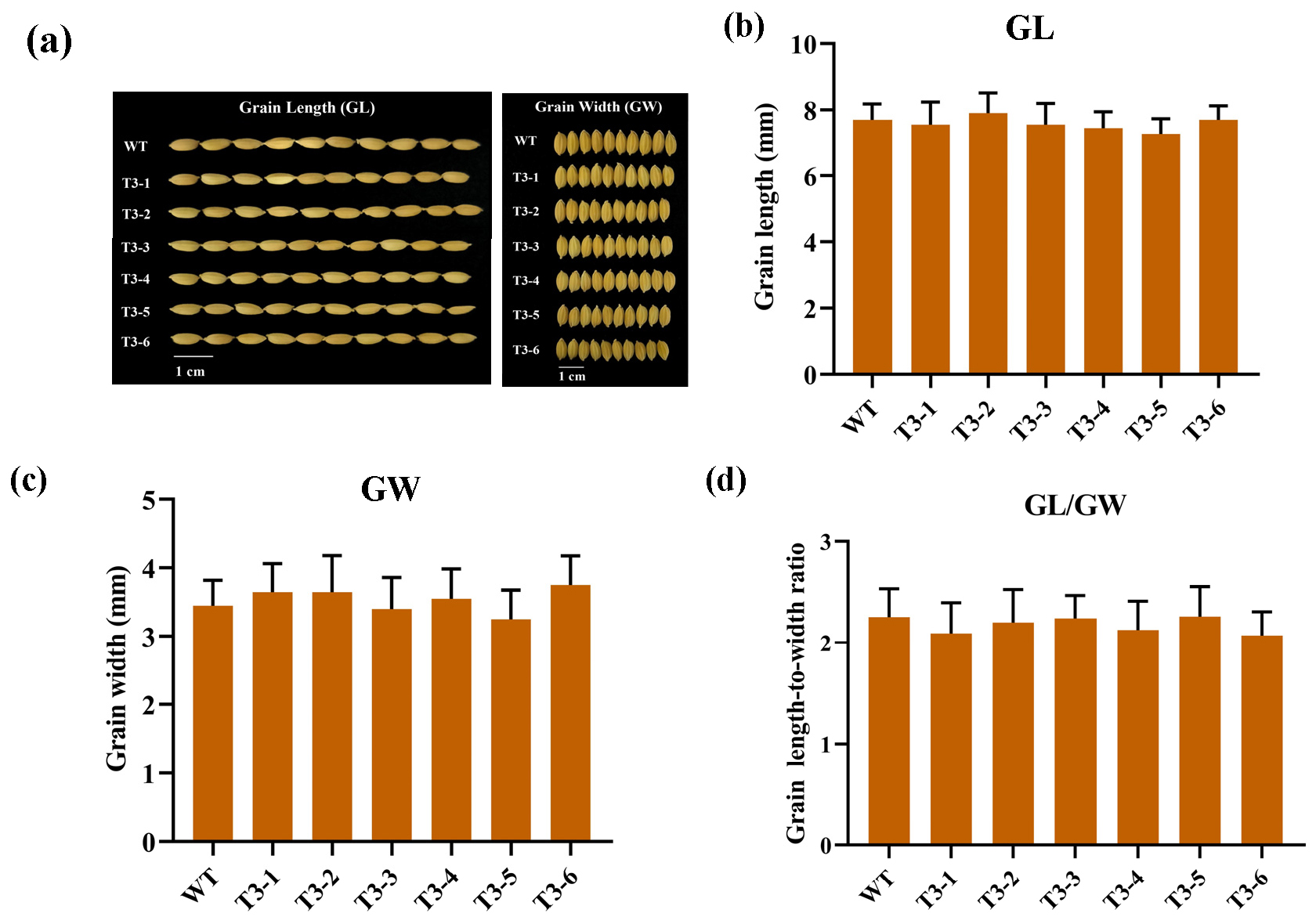
**

**Fig. S7 Effects of rS1 expression on grain size in transgenic rice.** Grains were randomly selected from T_3_ generation transgenic rice and wild type (WT) plants for size analysis. **(a)** Measurement of grain length and width. **(b)** Analysis of grain length (GL). **(c)** Analysis of grain width (GW). **(d)** Analysis of grain length/width ratio (GL/GW).


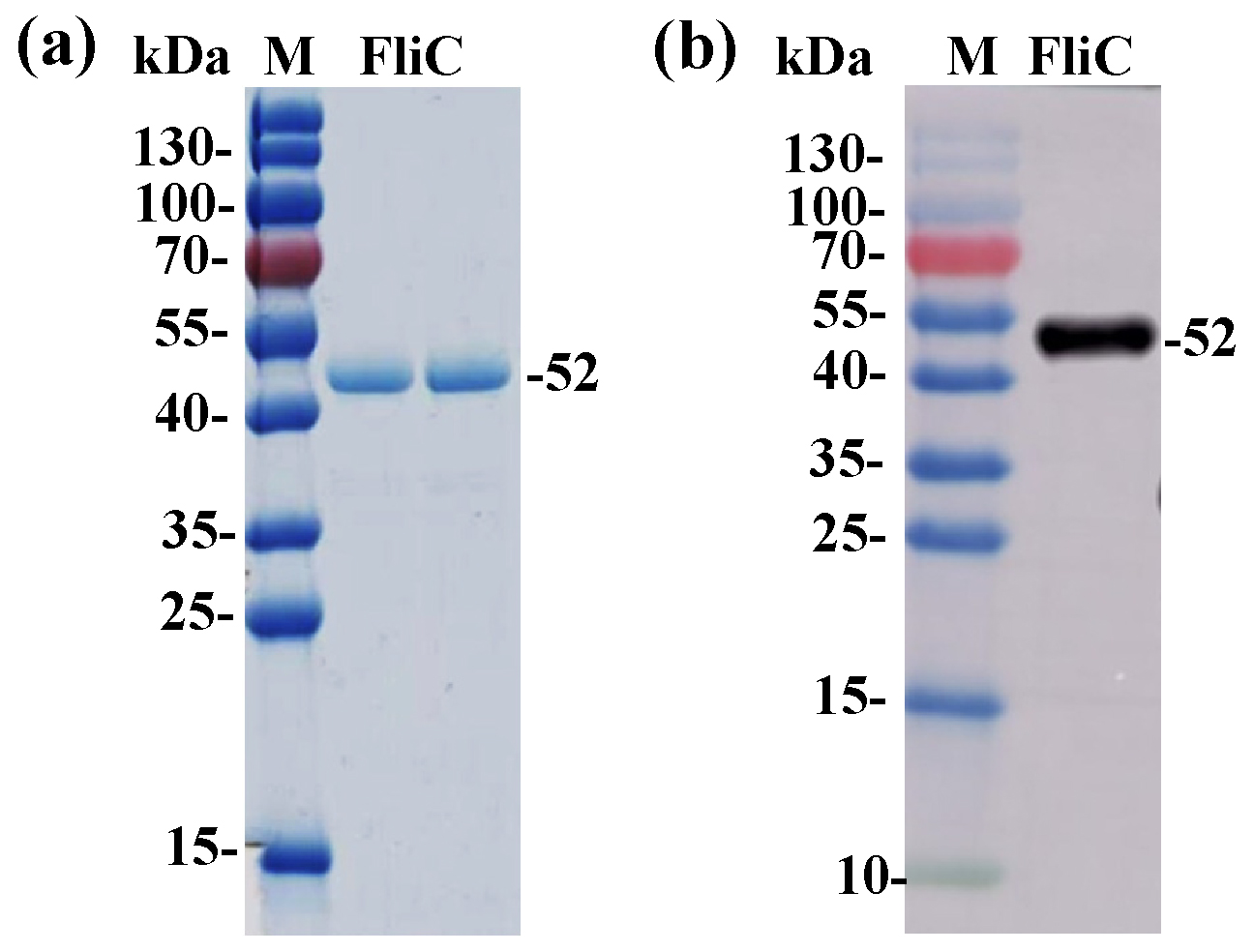
**Fig. S8**

**Fig. S8 Preparation and characterisation of *Salmonella* FliC adjuvant. (a)** SDS-PAGE analysis of FliC. Highly purified FliC was extracted from *Salmonella* Typhimurium ATCC14028s (pTrc99a-*fliC*-WT). **(b)** Immunoreactivity of the purified FliC protein was confirmed through Western blot analysis with an anti-FliC antibody.

**Fig. S9**

**
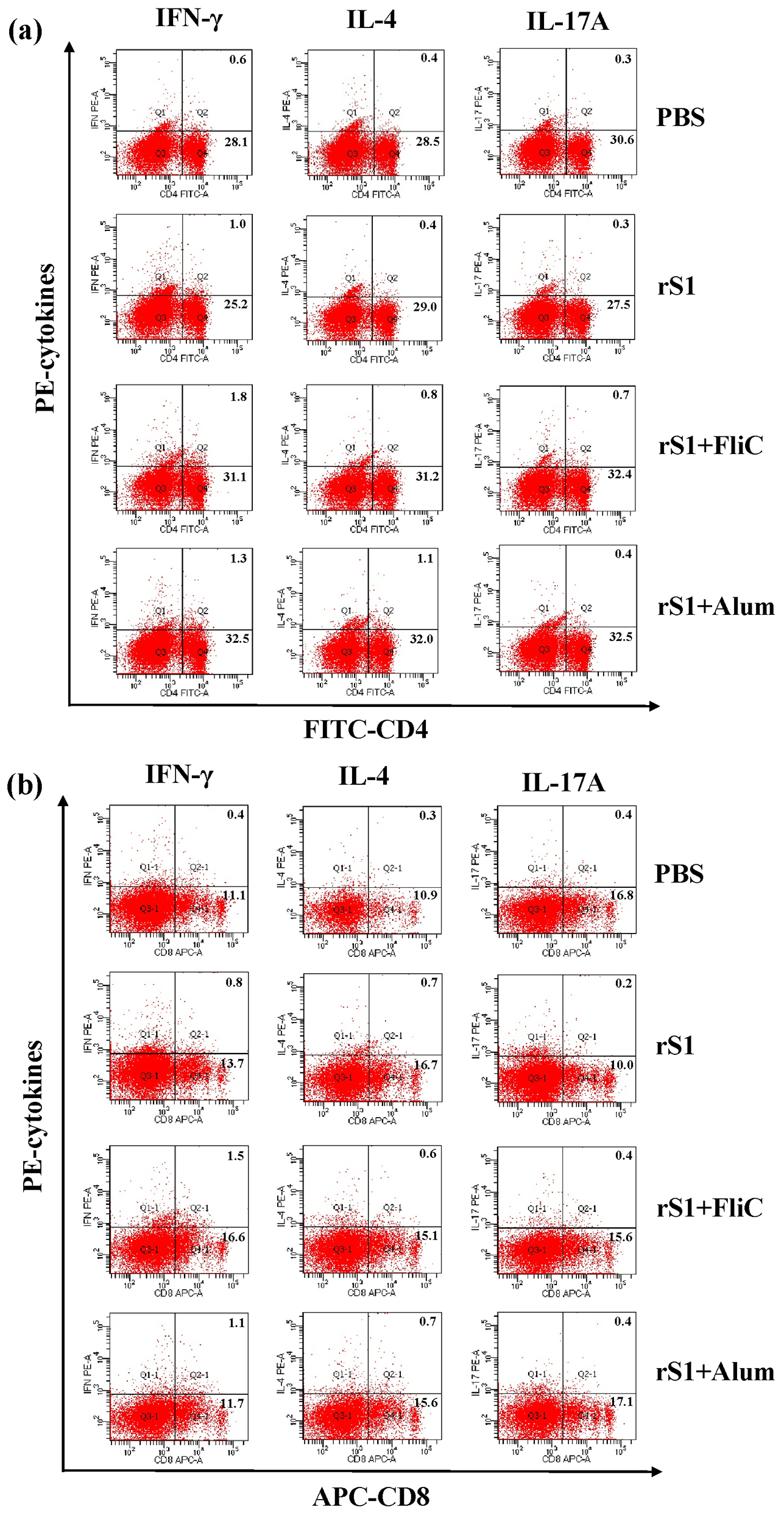
**

**Fig. S9 Illustration of cytokine-producing CD4^+^ and CD8^+^ T cells using flow cytometry.** Splenocytes were harvested from vaccinated mice two weeks after the last immunization and restimulated with the rS1 protein for 6 h. Cytokine-producing CD4**^+^** and CD8**^+^** T cell responses were detected by intracellular staining and quantified by flow cytometry. Flow cytometer plots of results obtained from one representative individual mouse in each group are presented. The percentages of the indicated cells are shown in the quadrant areas.
