# Supplementary material for: Rice‐derived SARS‐CoV‐2 glycoprotein S1 subunit vaccine elicits humoral and cellular immune responses
Source: Plant Biotechnol J. 2025 Apr 4;23(7):2570–82. doi: 10.1111/pbi.70077 (PMC12205891; doi:10.1111/pbi.70077)
Supplement: Supplementary file 2 — Table S1 Primers used for construction and identification of S1 gene. Table S2 Primer sequences for qRT‐PCR of cytokines. Table S3 PCR analysis of genomic DNA from transgenic rice lines. Table S4 Hygromycin‐resistant analysis of transgenic rice. [file PBI-23-2570-s001.docx]

**Table S1 Primers used for construction and identification of S1 gene**

| Primers | Sequence (5'-3') | Restricted site |
| --- | --- | --- |
| Gt1-S1F | TAGTCCTACAACAACGGATCCTTGTCTGATTGATCATCAAT | *Bam*H I |
| Gt1-S1R | CGATCGGGGAAATTCGAGCTCTCACAACTCATCTTTCCTCG | *Sac* I |
| Actin-S1F1 | GGTACCCGGGGATCCTCTAGATTGTCTGATTGATCATCACA | *Xba* I |
| Actin-S1F2 | TTGTCTGATTGATCATCACAGTGCGTTAATCTTACCACTCG | — |
| Actin-S1R | ATCCTTGTAATCTCCGTCGACCAACTCATCTTTCCTCGCGC | *Sal* I |
| S1-F1 | CAGTGCGTTAATCTTACCACTCGGAC | — |
| S1-R1 | CCTCGCGCGCCTAGGGCTGTTAGTCTG | — |
| S1-F2 | GAGACAAAATGCACGCTCAA | — |
| S1-R2 | CTCTTTCAAACGGCTTCAGG | — |
| qS1-F | GCTTCACCAACGTGTATGCT | — |
| qS1-R | TTCCCGCCCACCTTACTATC | — |
| qS1-P | AGCTCCTGGCCAAACCGGCA | — |

**Table S2 Primer sequences for qRT-PCR of cytokines**

| Gene | Primer sequences (5'-3') | Product size (bp) | Accession no. |
| --- | --- | --- | --- |
| IL-4 | F: TCACAGCAACGAAGAACACC | 151 | NM_021283.2 |
|  | R: CGAAAAGCCCGAAAGAGTC |  |  |
| IL-10 | F: ACCTGGTAGAAGTGATGCC | 194 | NM_010548.2 |
|  | R: GACACCTTGGTCTTGGAG |  |  |
| IFN-γ | F: ACTGGCAAAAGGATGGTGAC | 237 | NM_008337.4 |
|  | R: TGAGCTCATTGAATGCTTGG |  |  |
| TNF-α | F: AGCCCCCAGTCTGTATCCTT | 212 | NM_013693.3 |
|  | R: CTCCCTTTGCAGAACTCAGG |  |  |
| IL-17A | F: TCCAGAAGGCCCTCAGACTA | 239 | NM_010552.3 |
|  | F: AGCATCTTCTCGACCCTGAA |  |  |
| GAPDH | F: AACTTTGGCATTGTGGAAGG | 223 | NM_001289726.2 |
|  | R: ACACATTGGGGGTAGGAACA |  |  |

**Table S3 PCR analysis of genomic DNA from transgenic rice lines**

| pGt1::S1  Lines | Positive plant (%) | pActin::S1  Lines | Positive plant (%) | pGt1::S1  Lines | Positive plant (%) |
| --- | --- | --- | --- | --- | --- |
| T_1_-1 | 70 | T_1_-31 | 100 | T_2_-1 | 80 |
| T_1_-2 | 56 | T_1_-32 | 100 | T_2_-2 | 100 |
| T_1_-3 | 70 | T_1_-33 | 100 | T_2_-3 | 100 |
| T_1_-4 | 90 | T_1_-34 | 100 | T_2_-4 | 100 |
| T_1_-5 | 70 | T_1_-35 | 100 | T_2_-5 | 88 |
| T_1_-6 | 40 | T_1_-36 | 100 | T_2_-6 | 100 |
| T_1_-7 | 100 | T_1_-37 | 100 | T_2_-7 | 40 |
| T_1_-8 | 70 | T_1_-38 | 90 | T_2_-8 | 0 |
| T_1_-9 | 100 | T_1_-39 | 100 | T_2_-9 | 78 |
| T_1_-10 | 78 | T_1_-40 | 100 | T_2_-10 | 100 |
| T_1_-11 | 80 | T_1_-41 | 100 | T_2_-11 | 40 |
| T_1_-12 | 80 | T_1_-42 | 100 | T_2_-12 | 100 |
| T_1_-13 | 100 | T_1_-43 | 67 | T_2_-13 | 100 |
| T_1_-14 | 100 | T_1_-44 | 90 | T_2_-14 | 86 |
| T_1_-15 | 100 | T_1_-45 | 100 | T_2_-15 | 75 |
| T_1_-16 | 100 | T_1_-46 | 70 | T_2_-16 | 100 |
| T_1_-17 | 100 | T_1_-47 | 90 | T_2_-17 | 70 |
| T_1_-18 | 100 | T_1_-48 | 80 | T_2_-18 | 78 |
| T_1_-19 | 100 | T_1_-49 | 90 | T_2_-19 | 90 |
| T_1_-20 | 100 | T_1_-50 | 90 | T_2_-20 | 78 |
| T_1_-21 | 100 | T_1_-51 | 100 | **Total** | **80** |
| T_1_-22 | 70 | T_1_-52 | 100 |  |  |
| T_1_-23 | 100 | T_1_-53 | 100 | T_3_-1 | 100 |
| T_1_-24 | 100 | T_1_-54 | 100 | T_3_-2 | 100 |
| T_1_-25 | 100 | T_1_-55 | 100 | T_3_-3 | 100 |
| T_1_-26 | 100 | T_1_-56 | 90 | T_3_-4 | 100 |
| T_1_-27 | 100 | **Total** | **95** | T_3_-5 | 100 |
| T_1_-28 | 100 |  |  | T_3_-6 | 100 |
| T_1_-29 | 100 |  |  | **Total** | **100** |
| T_1_-30 | 100 |  |  |  |  |
| **Total** | **89** |  |  |  |  |

**Table S4 Hygromycin-resistant analysis of transgenic rice**

| pGt1::S1 Lines | Number of T4 plants  Control | | Number of T4 plants  Treatment | | H^R^ plants (%) |
| --- | --- | --- | --- | --- | --- |
|  | Germination (H-) | Growth  (H-) | Germination  (H-) | Growth  (H+) |  |
| T3-1 | 46 | 46 | 50 | 50 | 100 |
| T3-2 | 44 | 44 | 43 | 43 | 100 |
| T3-3 | 46 | 46 | 44 | 44 | 100 |
| T3-4 | 49 | 49 | 46 | 46 | 100 |
| T3-5 | 50 | 50 | 49 | 49 | 100 |
| T3-6 | 44 | 44 | 47 | 47 | 100 |
| T1-8 | 42 | 42 | 44 | 34 | 77 |
| WT | 50 | 50 | 47 | 0 | 0 |
